# Multispectral fluorescence imaging of EGFR and PD-L1 for precision detection of oral squamous cell carcinoma: a preclinical and clinical study

**DOI:** 10.1186/s12916-024-03559-w

**Published:** 2024-08-26

**Authors:** Nenghao Jin, Yu An, Yu Tian, Zeyu Zhang, Kunshan He, Chongwei Chi, Wei Mu, Jie Tian, Yang Du

**Affiliations:** 1 Medical School of Chinese PLA, Beijing, 100853 China; 2 Department of Stomatology, The First Medical Centre, Chinese PLA General Hospital, Beijing, 100853 China; 3 CAS Key Laboratory of Molecular Imaging, Beijing Key Laboratory of Molecular Imaging, Institute of Automation, Chinese Academy of Sciences, Beijing, 100190 China; 4 Key Laboratory of Big Data-Based Precision Medicine (Beihang University), Ministry of Industry and Information Technology of the People’ S Republic of China, School of Engineering Medicine, Beihang University, Beijing, 100191 China; 5 Department of Stomatology, Beijing Integrated Traditional Chinese and Western Medicine Hospital, Beijing, 100039 China; 6 University of Chinese Academy of Sciences, Beijing, 100080 China; 7 State Key Laboratory of Computer Science and Beijing Key Lab of Human-Computer Interaction, Institute of Software, Chinese Academy of Sciences, Beijing, 100190 China

**Keywords:** Oral squamous cell carcinoma (OSCC), Epidermal growth factor receptor (EGFR), Programmed death-ligand 1 (PD-L1), Multispectral fluorescence molecular imaging, Imaging probe

## Abstract

**Background:**

Early detection and treatment are effective methods for the management of oral squamous cell carcinoma (OSCC), which can be facilitated by the detection of tumor-specific OSCC biomarkers. The epidermal growth factor receptor (EGFR) and programmed death-ligand 1 (PD-L1) are important therapeutic targets for OSCC. Multispectral fluorescence molecular imaging (FMI) can facilitate the detection of tumor multitarget expression with high sensitivity and safety. Hence, we developed Nimotuzumab-ICG and Atezolizumab-Cy5.5 imaging probes, in combination with multispectral FMI, to sensitively and noninvasively identify EGFR and PD-L1 expression for the detection and comprehensive treatment of OSCC.

**Methods:**

The expression of EGFR and PD-L1 was analyzed using bioinformatics data sources and specimens. Nimotuzumab-ICG and Atezolizumab-Cy5.5 imaging probes were developed and tested on preclinical OSCC cell line and orthotopic OSCC mouse model, fresh OSCC patients’ biopsied samples, and further clinical mouthwash trials were conducted in OSCC patients.

**Results:**

EGFR and PD-L1 were specifically expressed in human OSCC cell lines and tumor xenografts. Nimotuzumab-ICG and Atezolizumab-Cy5.5 imaging probes can specifically target to the tumor sites in an in situ human OSCC mouse model with good safety. The detection sensitivity and specificity of Nimotuzumab-ICG in patients were 96.4% and 100%, and 95.2% and 88.9% for Atezolizumab-Cy5.5.

**Conclusions:**

EGFR and PD-L1 are highly expressed in OSCC, the combination of which is important for a precise prognosis of OSCC. EGFR and PD-L1 expression can be sensitively detected using the newly synthesized multispectral fluorescence imaging probes Nimotuzumab-ICG and Atezolizumab-Cy5.5, which can facilitate the sensitive and specific detection of OSCC and improve treatment outcomes.

**Trial registration:**

Chinese Clinical Trial Registry, ChiCTR2100045738. Registered 23 April 2021, https://www.chictr.org.cn/bin/project/edit?pid=125220

**Supplementary Information:**

The online version contains supplementary material available at 10.1186/s12916-024-03559-w.

## Background

Oral malignancies account for 3–5% of all malignant tumors. Oral squamous cell carcinoma (OSCC) is a kind of oral malignant tumor with high malignant degree and poor prognosis. In 185 countries around the world, there are about 476,000 new cases of OSCC and about 226,000 deaths [1]. The overall mortality rate of oral and oropharyngeal cancers has increased by 0.5% per year over the past 10 years [2]. After comprehensive treatment, the 5-year survival rate remains at 50%, and the survival rate of patients with advanced disease decreases to 27% [3, 4]. The poor prognosis of patients with OSCC is partially due to a delayed diagnosis. Early screening and timely treatment can effectively prevent the progression of OSCC, thereby increasing the survival rate of patients [5, 6]. Currently, the diagnostic methods for OSCC mainly involve visual inspection and tactile examination of the oral cavity, head and neck lymph nodes, and traditional medical imaging such as computed tomography (CT) and magnetic resonance imaging (MRI) [7]. However, some OSCCs appear similar to oral ulcers or underlying oral malignant diseases (OPMDs) [8, 9], leading to misdiagnosis. The gold standard for a definitive OSCC diagnosis is pathological examination, which is invasive, time-consuming, and involves complicated procedures [10–12]. Hence, there is an urgent need for new ways to detect tumors noninvasively and sensitively.

Near-infrared fluorescence (NIRF) imaging, through the injection or application of fluorescent imaging probes, allows tumors to be fluorescently lightened in the corresponding diseased areas, providing precise guidance for discriminating healthy and tumor tissues safely [13]. The most common NIR dyes used for in vivo NIRF tumor imaging are indocyanine green (ICG), Cy5, Cy5.5, Cy7 and IRDye800CW [14]. ICG is the amphiphilic small-molecule NIRF dye approved by the food and drug administration (FDA) for the surgical treatment of head and neck tumors [15]. ICG was first used for NIRF imaging in 9 patients with head and neck squamous cell carcinoma (HNSCC) [16]. Bredell et al. detected cervical sentinel lymph nodes in the soft tissue after peritumoral injection of ICG in patients with oropharyngeal cancer [17]. However, free ICG and other NIR dyes are non-specific and cannot be used for tumor-targeted imaging. NIR dyes can be coupled with tumor-specific ligands such as antibodies, metabolic substrates, cell surface peptides, and growth factors to recognize tumor cells [18–21]. In HNC, Cetuximab-800CW was evaluated by fluorescence-guided imaging (FGI) for high sensitivity to tumor detection [22, 23]. Multispectral fluorescence imaging (FMI) mainly uses broad-spectrum light and can simultaneously detect multiple biomarkers expressed in tumors. Chen et al. demonstrated the feasibility of detecting multiple targets in human for the first time in Barrett's neoplasia study. Multispectral FMI can facilitate precise and early detection of tumors [24]. Compared with target-FMI with a single tracer, multispectral FMI can improve the sensitivity and specificity of tumor identification [25]. For localized, small-scale tumors, the extracellular matrix (ECM) may prevent the penetration of intravenously administered macromolecules, leading to missed diagnoses of small lesions [26]. The use of micro-dose fluorescent imaging probes to spray or smear the diseased area enables almost instant imaging [27]. For OSCC, the entire oral mucosa can be topically applied with targeted imaging probes, and suspected cancer lesions can be directly lightened and correspondingly detected [28–32].

The epidermal growth factor receptor (EGFR) and programmed death-ligand 1 (PD-L1) are two key important therapeutic targets for OSCC. Targeted therapy, represented by EGFR antibody drugs, and immunotherapy, represented by anti-PD-1/PD-L1 antibodies, have provided new strategies for the treatment of HNSCC. Cetuximab, an anti-EGFR monoclonal antibody (mAb), was the first FDA-approved molecular-targeted drug for the treatment of HNSCC [33] and has been approved as a first-line treatment for relapsed and/or metastatic HNSCC. Fluorescently labeled cetuximab can be utilized for targeted FMI of OSCC to evaluate the targeted recognition of anti-EGFR fluorescently labeled antibodies in and around the tumor after systemic administration [34]. de Wit JG et al. [23] reported that Cetuximab-800CW can be used for the detection of positive tumor margins through 66 OSCC tumors and can safely locate surgical margins during surgery. Nimotuzumab has a lower affinity than Cetuximab, and has less effect on the recognition and killing of normal tissue cells [35]. PD-L1 is a transmembrane receptor that, after binding to PD-L1/PD-L2, can prevent cellular immunity. PD-L1 antibody drugs can relieve this inhibition and enhance the cellular immune response against tumor cells by blocking the PD-1/PD-L1 signaling pathway [36]. The potential for imaging with Atezolizumab and its ability to predict responses to PD-L1 immunotherapy were initially assessed in a human study using zirconium-89-labeled Atezolizumab (anti-PD-L1) [37]. Many cancers, including OSCC, are molecularly heterogeneous, and the detection of multiple targets is needed for more accurate clinical diagnosis and guidance for personalized therapy.

**Fig. 1:**
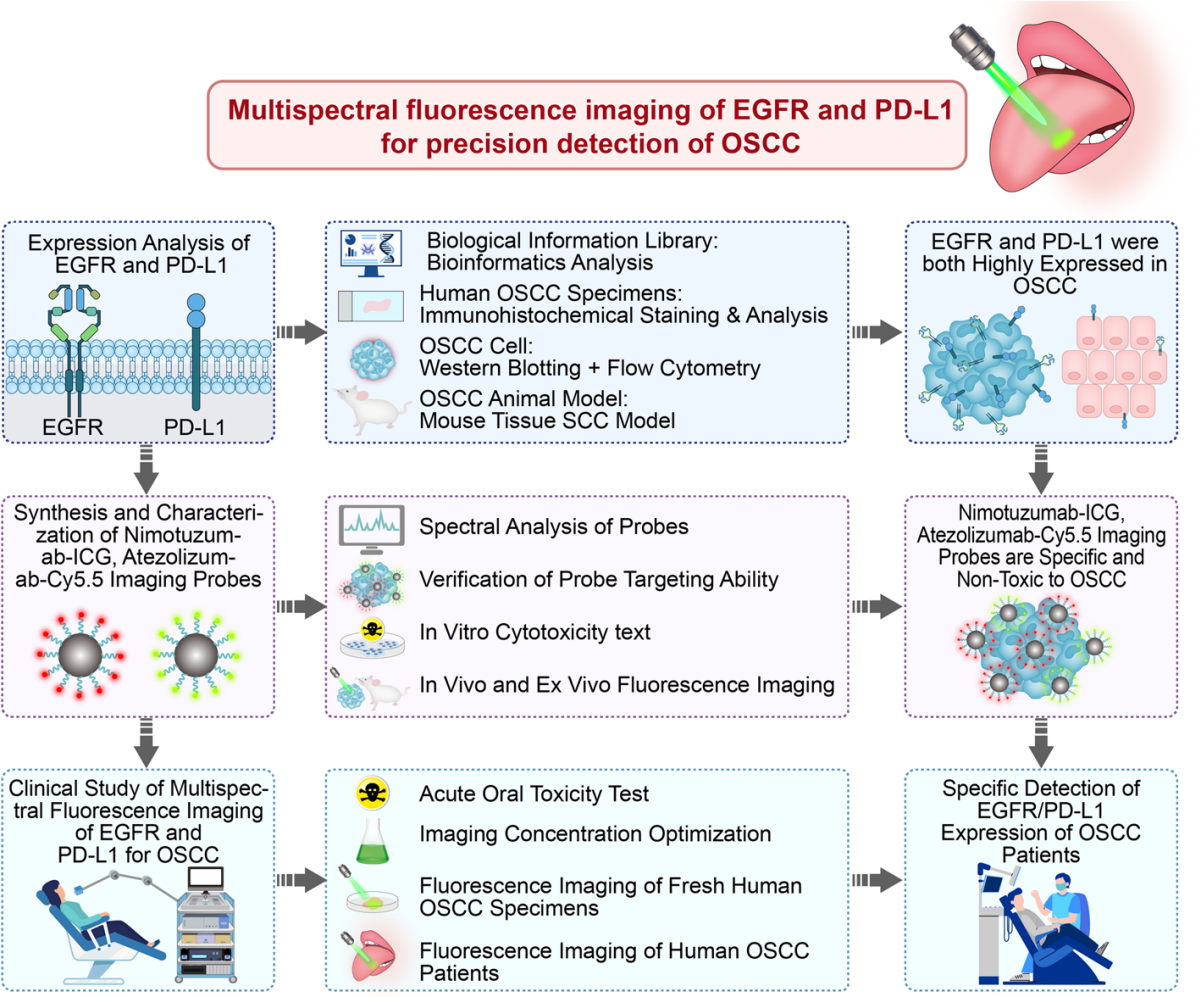
Study flowchart

Hence, the aims of this study is to validate EGFR and PD-L1 as specific biomarkers for OSCC and develop multispectral Nimotuzumab-ICG and Atezolizumab-Cy5.5 fluorescence imaging probes to quantify the dynamic expression of dual-target EGFR and PD-L1 from preclinical in vitro human OSCC cell lines, in vivo OSCC orthotopic mouse models, to clinical OSCC patient tumor specimens, and topical application as mouthwash in OSCC patients. The new imaging strategies, quick and sensitive detection methods, and experimental evidence for the sensitive detection of OSCC biomarkers provided in this study will help to provide guidance for the early detection of OSCC, targeted therapy and immunotherapy (Fig. 1).

## Methods

### Bioinformatics analysis

The data used to compare EGFR and PD-L1 expression in HNSCC and normal tissues are available in the gene expression profiling interactive analysis (GEPIA) database at http://gepia.cancer-pku.cn.

### Clinical model prediction based on receiver operator characteristic (ROC) analysis

One hundred and twelve OSCC patients treated at the Chinese PLA General Hospital between December 2018 and December 2019 were retrospectively analyzed. The inclusion criteria were patients who underwent surgery for the first time at our hospital and were diagnosed with OSCC based on complete clinical and follow-up records. Exclusion criteria: (1) presence of other concomitant tumors; (2) presence of distant metastases; (3) history of preoperative antineoplastic therapy; (4) serious postoperative complications. The follow-up period was three years, and the patients were re-examined every three months. The time of recurrence, metastasis, or death was defined as the cutoff time. The follow-up period ended on December 1, 2022. Receiver operating characteristic (ROC) curve analysis was performed based on EGFR and PD-L1 expression. EGFR and PD-L1 expression was evaluated by two independent pathologists in our hospital who were blinded to the study design. EGFR expression was detected using immunohistochemistry (IHC). The negative, weak positive, moderate positive, strong positive staining were respectively defined as —, + , +  + , +  +  + . PD-L1 expression was measured using PD-L1 IHC 22C3 pharmDx (Dako 22C3, Dako, Denmark) and ranged from 0 to 99% as determined using the Tumor Proportion Score (TPS).

### EGFR and PD-L1 expression analysis via IHC of human OSCC

Analyses of EGFR and PD-L1 expression were conducted on biospecimens from the Department of Stomatology, First Medical Center, Chinese PLA General Hospital. All biopsy specimens were diagnosed as OSCC by the Department of Pathology. The biospecimens were then embedded and sliced continuously. Tissue sections were incubated with bovine albumin (BSA, GC305010; Servicebio, Wuhan, China) for 30 min and Rabbit monoclonal antibody to EGFR (RRID: AB_869579; Abcam, Cambridge, UK) and Rabbit monoclonal antibody to PD-L1 (RRID: AB_2687878; Abcam, Cambridge, UK) at 4 °C overnight. The slides were then exposed to HRP peroxidase-labeled Goat Anti-Rabbit IgG (RRID: AB_2811189; Servicebio, Wuhan, China) and incubated for 1 h. Subsequently, the sections were stained with hematoxylin and eosin (H&E) for analysis. The expression levels of EGFR and PD-L1 were quantified using H-scores (0–300). Images were then captured using a scanner (Digital Sight DS-U3, Eclipse E100, Nikon, Japan), and H-scores were calculated by ImageJ Fiji (NIH, Bethesda, MD, USA).

### Cell culture

Normal human oral keratinocytes (HOK) (ScienCell Research Laboratories Inc., USA) cells were cultured in Roswell Park Memorial Institute 1640 (RPMI-1640) medium (Thermo Fisher Scientific) containing 10% fetal bovine serum (FBS) (Thermo Fisher Scientific Australia Pty Ltd., Australia) and 1% penicillin–streptomycin (Macgene Biotechnology, China). Human OSCC CAL27 cells (RRID: CVCL_1107; ATCC, Manassas, VA, USA), CAL27-Fluc cells (RRID: CVCL_1107; Cobioer Biosciences Co., Ltd., China) and HSC3 cells ((RRID: CVCL_1288; ATCC, Manassas, VA, USA) were cultured in Dulbecco’s modified Eagle’s medium (DMEM) (Thermo Fisher Scientific (CHINA) Co., Ltd., China) containing 10% FBS and 1% penicillin–streptomycin. All cells were cultured in a Cell Culture Dish (Corning Inc., USA). All experiments were performed using mycoplasma-free cells. All human cell lines were authenticated using STR profiling within the last three years.

### EGFR and PD-L1 expression analysis in different cells via western blotting and flow cytometry

HOK, CAL27, CAL27-Fluc and HSC3 cells were cultured and collected. Total protein was extracted from cells using precooled Pierce RIPA Buffer (Thermo Fisher Scientific, USA) and Halt Protease Inhibitor Cocktail (Thermo Fisher Scientific, USA), and western blotting was conducted utilizing Rabbit monoclonal antibody to EGFR (RRID: AB_869579) and PD-L1 monoclonal antibody (RRID: AB_2756526; Proteintech Group, Inc., USA), polyclonal rabbit β-actin antibody (Cell Signaling Technology, USA), and secondary antibody (1:10,000, Jackson ImmunoResearch, USA) were used for western blot. All four cell types (1 × 10^6^) were incubated with FITC labeled Anti-EGFR antibody (RRID:AB_298005) and APC Anti-PD-L1 antibody (ab206967, Abcam, Cambridge, UK). After 30 min incubation, the cells were suspended in Phosphate-Buffered Saline (PBS) (Thermo Fisher Scientific Co., Ltd., China) and analyzed using flow cytometry (FACSCanto™ II; BD Biosciences, USA). FlowJo (version10.8.1) software was used for data analysis. The experiment was repeated three times, and the average of the results was taken.

### Human OSCC orthotopic mouse model

All animal experiments were approved according to the guidelines of the Institutional Animal Care and Use Committee (Permit No: IA21-2203–24) of the Institute of Automation, Chinese Academy of Sciences. PBS (50 μL) containing 5 × 10^6^ HSC3 or CAL27-Fluc cells were injected into the tongues of 6-week-old male BALB/c-nu/nu mice anesthetized with isoflurane (RWD Life Science, China). The feeding method is to give soft food or softened food with feed water. HSC3 and CAL27-Fluc tumor xenografts were harvested 7–10 days after inoculation; H&E staining was performed. Primary anti-EGFR antibody (RRID: AB_869579) and anti-PD-L1 antibody (RRID: AB_2687878) were used for immunohistochemical and immunofluorescence staining to analyze the expression of EGFR and PD-L1 in human OSCC tumor xenograft mouse models.

### Synthesis of Nimotuzumab-ICG and Atezolizumab-Cy5.5

ICG-NHS-ester (330 nM, dissolved in 300 μL DMSO, Sigma-Aldrich) was added to humanized monoclonal EGFR antibody (5 mg, 33 nM, Nimotuzumab, Biotech Pharma, China) (dye/antibody ratio was 1:20) in conjugation buffer (5 mL, 0.1 M PBS), and the solution was reacted in the dark at 25 °C with continuous oscillation overnight. The mixture was concentrated to 0.8 mL using a centrifugal filter unit (3 kDa MWCO, Amicon) and purified three times via centrifugation at 8,500 rpm for 15 min. Similarly, Cy5.5-NHS-ester (330 nM, dissolved in 300 μL 0.1 M PBS, New Research Bioscience, China) was added to humanized monoclonal anti-PD-L1 antibody (Atezolizumab, Roche Registration GmbH, Switzerland) in conjugation buffer (5 mL, 0.1 M PBS), and the solution was reacted in the dark at 25 °C with continuous oscillation overnight. The mixture was concentrated and purified thrice using a centrifugal filter unit (3 kDa MWCO, Amicon). The conjugation efficiency was calculated.

### In vitro cell viability experiments and spectroscopic analysis

Logarithmic growth-phase HOK, CAL27-Fluc, and HSC3 cells were seeded (5 × 10^3^/well) in 96-well culture plates (Corning Inc., USA) overnight. Nimotuzumab-ICG, Atezolizumab-Cy5.5 and the mixture were added into the culture plates at different concentrations (2.5, 5, 10, 20, 40, 80, 160, and 320 μg/mL), respectively. After 24 h incubation, cells were washed thrice with PBS and cultured in 10% CCK-8 medium (CA1210; Solarbio Science and Technology Co., Ltd., China) for 30 min; the absorbance at 450 nm was determined using a spectrophotometric microplate reader (Synergy HT; BioTek, USA).

The spectra of excitation light and absorption light of Nimotuzumab-ICG and Atezolizumab-Cy5.5 were tested by fluorescence spectrophotometer (F-7000, Hitachi, Japan) (The scanning speed is 1200 nm/min, the spectral bandwidth is 10 nm, and the sampling interval is 0.2 nm) and near-infrared spectrophotometer (UV-3600 Plus, Shimadzu, Japan), respectively.

### In Vivo and Ex vivo fluorescence molecular imaging (FMI) and safety evaluation

CAL27-Fluc OSCC tumor-bearing mice were injected with Nimotuzumab-ICG (0.2 mg) and Atezolizumab-Cy5.5 (0.2 mg), and their mixture via the caudal vein. The block group (normal mice) was intraperitoneally injected with Nimotuzumab (0.2 mg) and Atezolizumab (0.2 mg) 1 h before the intravenous injection of the imaging probes. FMI images were captured at different time points (pre-, 2, 6, 12, and 48 h) using an IVIS® Spectrum/CT (Caliper Life Sciences, USA), and the tumor to background ratio (TBR) was calculated using the region of interest (ROI) measurement tool with Fiji software.

After 24 h, the mice were sacrificed, and the serum were collected for detection of liver function indices, including alanine aminotransferase (ALT) and aspartate aminotransferase (AST) using an automatic blood biochemical auto analyzer (7600; Hitachi, Tokyo, Japan). The tongues, hearts, livers, spleens and kidneys were collected for ex vivo FMI by IVIS® SpectrumCT. H&E staining was performed on organs after ex vivo imaging. Resected fresh CAL27-Fluc tumor-bearing mouse tongue tumor specimens were analyzed using immunofluorescence (IF) staining for EGFR and PD-L1 expression.

### Probe fluorescence imaging of fresh human specimens

First, 10 fresh OSCC specimens were pre-tested to determine the optimal incubation concentration of the probe, and then a formal fluorescence imaging experiment on 20 OSCC specimens was followed.. A total of 30 resected fresh human OSCC specimens were rinsed in 5% goat serum (SL038, Solarbio Science and Technology Co., Ltd., China) to remove debris and mixed and incubated with Nimotuzumab-ICG and Atezolizumab-Cy5.5 at 20 °C for 3 min. Subsequently, the specimens were washed with PBS 3 times. A homemade multispectral fluorescence imaging system (Key Lab of Molecular Imaging, CAS) was used to perform imaging experiments on the specimens. The device can achieve optical resolution of 50 μm, bandwidth of 5 nm, imaging field of 12.5 × 12.5 cm^2^, 50 fps time resolution (adjustable according to actual conditions), real-time imaging of white light image, near-infrared dual imaging channels of 665 nm and 785 nm simultaneously. After imaging experiments, the specimens were fixed in 4% paraformaldehyde (P1110; Macgene Biotechnology) for subsequent H&E, EGFR, and PD-L1 IHC and IF staining.

### Fluorescence imaging of OSCC in human patients

This study was approved by the Ethics Committee of the Chinese PLA General Hospital (No. S2021-097–01) and registered in the Chinese Clinical Trial Registry (Registration Number: ChiCTR2100045738). All human patients signed informed consent, and the patients gargled 5 mL of Nimotuzumab-ICG (0.4 mg/mL) and Atezolizumab-ICG (0.4 mg/mL) for approximately 30 s. Next, the patients gargled ultrapure water for 30 s three times, and the interval between Nimotuzumab-ICG and Atezolizumab-ICG imaging was 1 h. White-light and fluorescence images were obtained using a commercialized Digital Precision Medicine imaging device H2800 (DPM, Beijing, China). After surgical treatment, fresh specimens were obtained for the probe-spraying experiment, H&E staining, EGFR, PD-L1 IHC staining, and IF staining. EGFR and PD-L1 expression were defined as —, + , +  + , +  +  + for negative, weak positive, medium positive, and strong positive expression.

### Statistical analysis

The optimal cutoff values for the expression of EGFR and PD-L1 were determined from the ROC curves. Statistical analyses were performed using SPSS (version 21.0; IBM, Armonk, NY, USA) and GraphPad Prism software (version 9; GraphPad Software Inc., San Diego, CA, USA). Differences between paired data were tested using the Wilcoxon test. Statistical significance was set as *P < 0.05, ** *P* < 0.01, ***P < 0.001, **** *P* < 0.0001.

## Results

### Validation of EGFR and PD-L1 as specific OSCC biomarkers

**Fig. 2:**
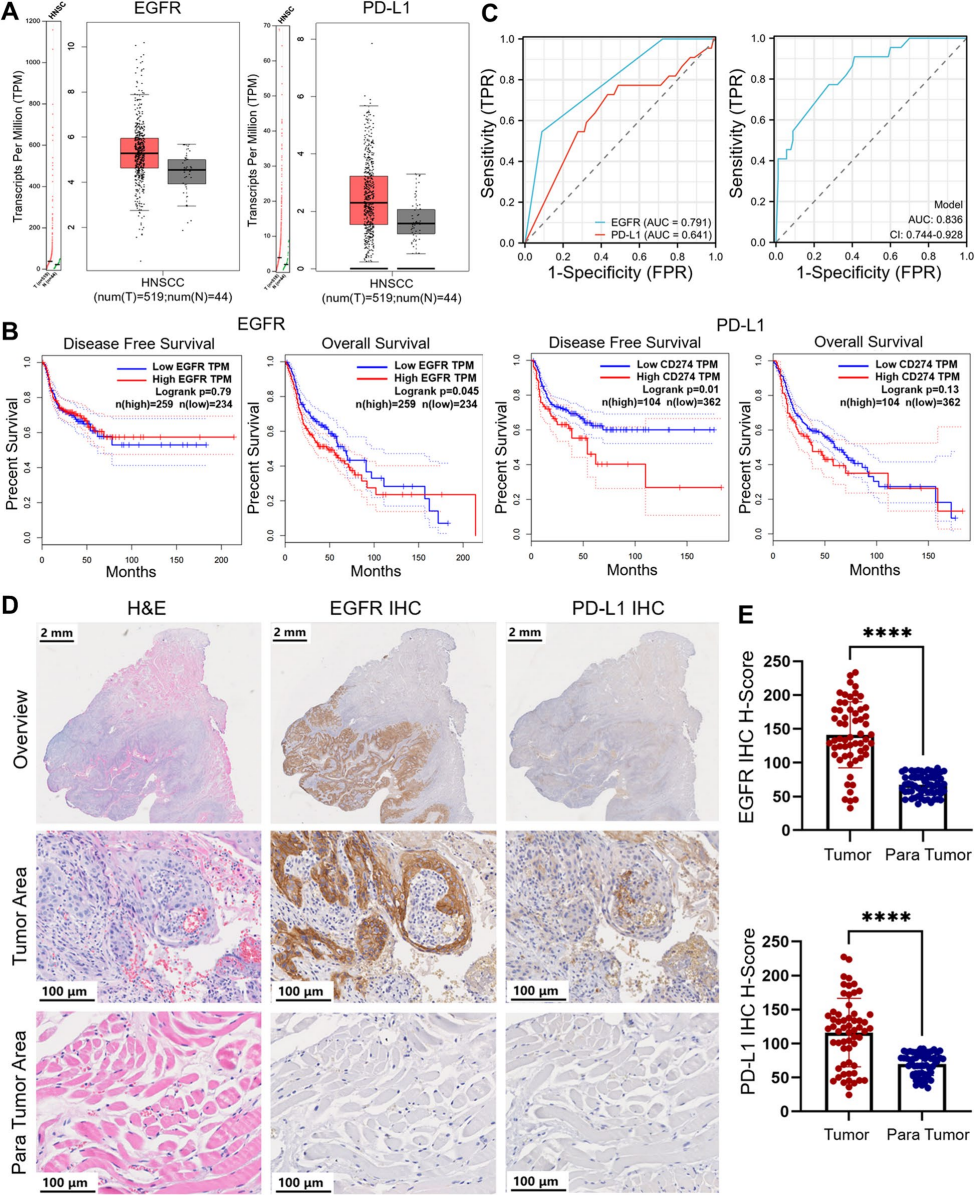
Expression and analysis of EGFR and PD-L1 in HNSCC in GEPIA database and OSCC patients. **A** Statistical analysis of EGFR and PD-L1 expression in HNSCC tumors and normal tissues obtained from the GEPIA database. Log2(TPM + 1) was used for the log-scale. HNSCC, Head and Neck Squamous Cell Carcinomas. **B** The correlation of EGFR and PD-L1 expression related to disease free survival (DFS) and overall survival (OS). **C** Receiver operating characteristic analysis of EGFR and PD-L1. FPR, False Positive Rate; TPR, True Positive Rate; AUC, Area Under Curve. **D** Representative EGFR and PD-L1 IHC and H&E histology obtained from OSCC biospecimens. Scale bar of overview, 2 mm. Scale bar of tumor and para tumor, 100 μm. (E) IHC H-scores of EGFR and PD-L1 expression in IHC samples (*n* = 59 patients). **** *p* < 0.0001

According to the GEPIA dataset, the expression levels of EGFR and PD-L1 were significantly higher in HNSCC tumors than para tumor tissues (Fig. 2A). The EGFR and PD-L1 gene expression profiles across all HNSCC tumor samples (*n* = 519) and paired normal tissues (*n* = 44) were expressed as log2[transcripts per million (TPM) + 1]]. Patients with OSCC had high EGFR and PD-L1 expression. The expression of EGFR and PD-L1 is associated with the prognosis of patients with HNSCC. The correlation between EGFR expression and disease-free survival (DFS) and overall survival (OS) in 493 patients with HNSCC, and PD-L1 expression, DFS, and OS in 466 patients with HNSCC were analyzed using the GEPIA dataset. EGFR overexpression was significantly associated with OS (*P = 0.045), whereas high expression of PD-L1 was associated with shorter DFS (*P = 0.01) (Fig. 2B).

The statistical data for OSCC patients (n = 112) are shown in Supplementary Table 1. The areas under the curve corresponding to high expression of EGFR and PD-L1 were 0.791 and 0.641, respectively, but the corresponding curve area for the combined prediction is 0.836 (95% Confidence Interval (*CI*): 0.744 to 0.928), suggesting that the combination of EGFR and PD-L1 dual-targets is important for the precise prognosis of OSCC (Fig. 2C).

To confirm that EGFR and PD-L1 are specific biomarkers for OSCC, biospecimen slides from patients with OSCC (*n* = 59) were immunohistochemically stained with anti-EGFR and anti-PD-L1 antibodies. The demographics of patients with OSCC (*n* = 59) are shown in Additional file 1: Table S1. EGFR and PD-L1 expression levels were distinctly higher in OSCC tumors than in para tumor tissues (Fig. 2D). We clearly distinguished tumor tissues from normal tissues by immunohistochemical staining for EGFR and PD-L1. The EGFR H-Score and PD-L1 H-Score in OSCC and para tumor tissues were 141.2 vs. 67.19 and 116.2 vs. 69.66, respectively (**** *P* < 0.0001) (Fig. 2E).

### Detection of EGFR and PD-L1 expression in in vitro OSCC cells and in vivo orthotopic OSCC tumor model

**Fig. 3:**
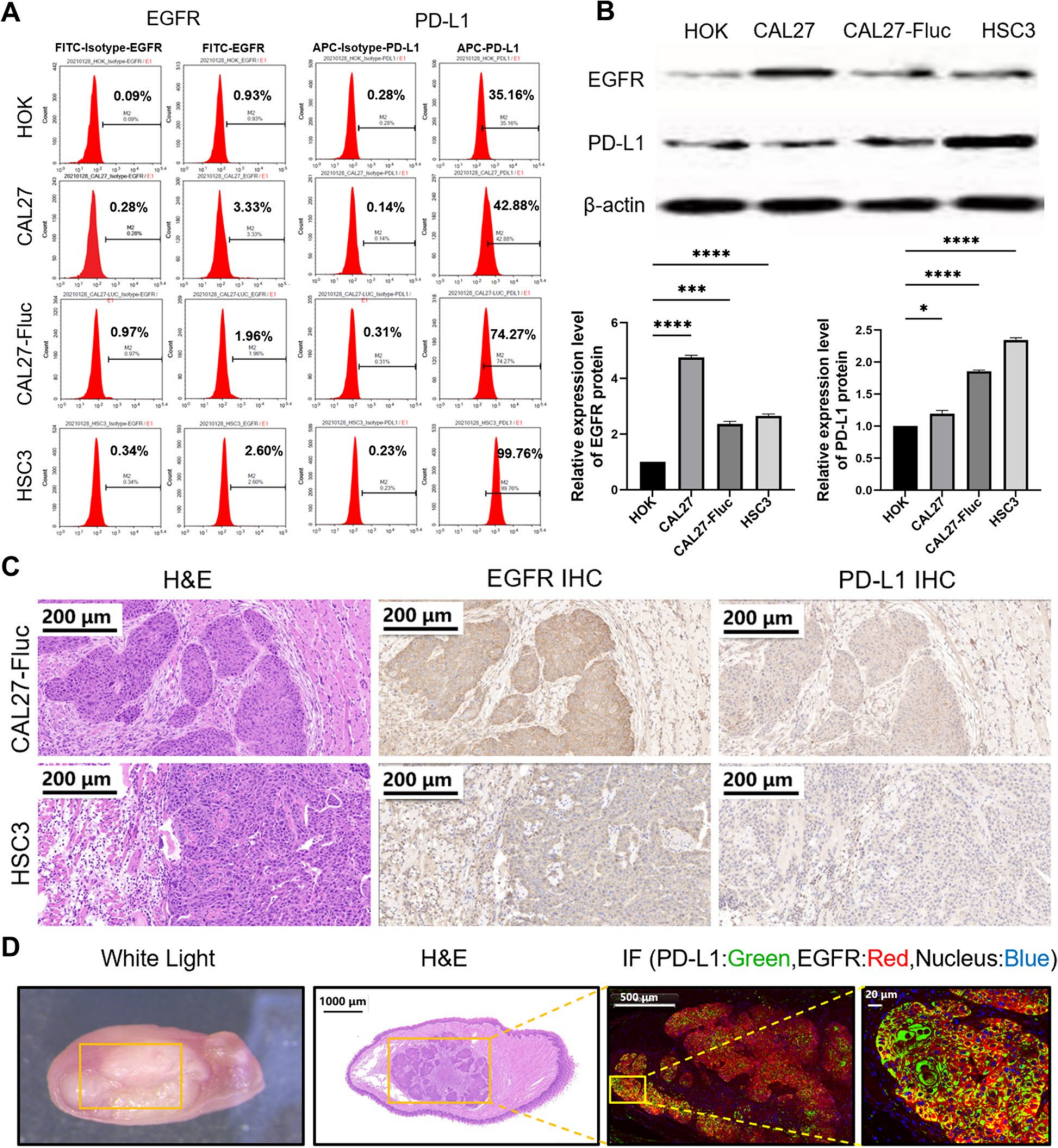
Detection of EGFR and PD-L1 Expression in in vitro OSCC cells and in vivo orthotopic OSCC tumor model. **A** FACS analysis of HOK, CAL27, CAL27-fLUC, and HSC3 cells for detection of EGFR and PD-L1 expression. **B** Western blotting analysis of EGFR and PD-L1 expression in HOK, CAL27, CAL27-fLUC, and HSC3 cell lines. *P < 0.05, ***P < 0.001, ****P < 0.0001. **C** H&E, EGFR and PD-L1 IHC staining of OSCC tumor xenograft. Scale bar, 200 μm. **D** White light, H&E, immunofluorescence staining (IF) obtained from CAL27-fLUC tumor xenografts. H&E Scale bar, 1,000 μm. Both PD-L1 and EGFR are over-expressed in the cell membrane and stained in green and red, respectively; the nucleus is stained in blue. Scale bar, 500 and 20 μm

The expression of EGFR and PD-L1 was relatively higher in CAL27, CAL27-Fluc, and HSC3 cells than in HOK cells, used as control (Fig. 3A). EGFR and PD-L1 protein expression was significantly higher in CAL27 (~ 4.75-fold, **** *P* < 0.0001, ~ 1.19-fold, * *P* < 0.05), CAL27-Fluc (~ 2.36-fold, *** *P* < 0.001, ~ 1.85-fold, **** *P* < 0.0001), and HSC3 (~ 2.65-fold, **** *P* < 0.0001, ~ 2.34-fold, **** *P* < 0.0001) cells than in HOK cells (Fig. 3B). IHC staining showed that EGFR and PD-L1 were both highly expressed in the tumor area in both CAL27-Fluc and HSC3 mouse models (Fig. 3C). EGFR and PD-L1 double IF staining also confirmed their high expression in the CAL27-Fluc mouse tumor xenografts (Fig. 3D). In general, our experiments confirmed that EGFR and PD-L1 were expressed at relatively higher levels in OSCC tumor tissues than in normal tissues.

### Characterization of Nimotuzumab-ICG and Atezolizumab-Cy5.5 imaging probes

Nimotuzumab and Atezolizumab were reacted with ICG-NHS and Cy5.5-NHS to yield Nimotuzumab-ICG and Atezolizumab-Cy5.5, respectively (Additional file 1: Fig. S1). The conjugation efficiency was 93.42% and 92.02%, respectively. Nimotuzumab-ICG had an excitation wavelength of 808 nm and absorption peak at 790 nm, which were partially offset from those of free ICG (Excitation: 815 nm), indicating that the dye was conjugated to Nimotuzumab. The spectral characterization of Atezolizumab-Cy5.5 also showed a shift from the excitation wavelength of free Cy5.5 (Excitation: 691 nm), indicating that the dye was linked to Atezolizumab, with excitation and absorption peak of Atezolizumab-Cy5.5 were 670 nm and 679 nm, respectively (Additional file 1: Fig. S2). The cell viability of different human OSCC cells was not influenced when the concentrations of Nimotuzumab-ICG and Atezolizumab-Cy5.5, were lower than 320 μg/mL. These results suggest that Nimotuzumab-ICG and Atezolizumab-Cy5.5 were relatively biocompatible and safe for further in vivo study (Additional file 1: Fig. S3).The in vivo toxicity of Nimotuzumab-ICG and Atezolizumab-Cy5.5 was evaluated in healthy male BALB/c-nu/nu mice; The mice were euthanized 24 h post-injection of PBS, Nimotuzumab-ICG, Atezolizumab-Cy5.5, and Nimotuzumab-ICG and Atezolizumab-Cy5.5 injection (4 mg/kg). Serum assessment showed that the ALT and AST levels were within the normal range in all four groups (Additional file 1: Fig. S4). In addition, no obvious histopathological changes were observed in the major organs (heart, liver, kidney, or spleen) (Additional File 1: Fig. S5). Nimotuzumab-ICG and Atezolizumab-Cy5.5 were further tested for in vivo transoral toxicity and proved to be safe (Additional file 1: Table S2). These results demonstrated that Nimotuzumab-ICG and Atezolizumab-Cy5.5 were well-developed and safe.

### The in vivo biodistribution and tumor targeting of Nimotuzumab-ICG and Atezolizumab-Cy5.5

**Fig. 4:**
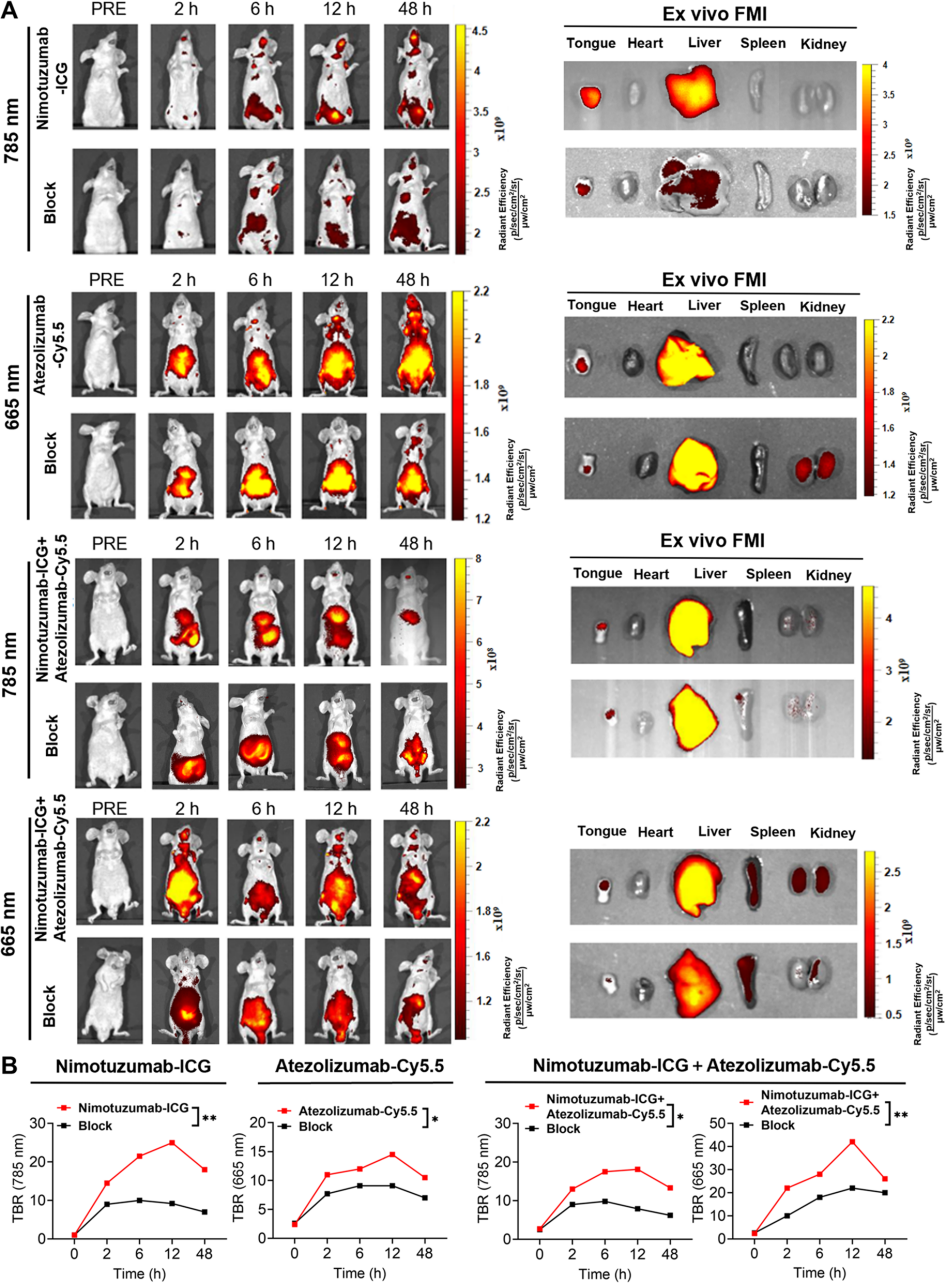
Biodistribution of Nimotuzumab-ICG and Atezolizumab-Cy5.5 in an orthotopic OSCC mouse model. **A** In vivo FMI of an OSCC orthotopic mouse model injected with Nimotuzumab-ICG, Atezolizumab-Cy5.5, or a combination of both, respectively. The blocking group represents the pre-administration of corresponding antibody drugs Ex vivo FMI of the tongue with OSCC tumor and major organs 48 h post-injection. **B** TBR analysis of in vivo FMI signal intensity in different groups at different time points. The TBR was significantly different at 12 h postinjection. FMI, fluorescence molecular imaging; TBR, tumor to background ratio. *P < 0.05, **P < 0.01

An orthotopic human CAL27-Fluc OSCC tumor mouse model was established and bioluminescence imaging (BLI) was performed to locate the OSCC tumor xenograft (Additional file 1: Fig. S6). CAL27-Fluc oral tumor-bearing mice were divided into different groups: Nimotuzumab-ICG, Atezolizumab-Cy5.5, Nimotuzumab-ICG + Atezolizumab-Cy5.5, and corresponding blocking groups. The fluorescence signals of ICG and Cy5.5 were detected at 785 nm and 665 nm, respectively (Fig. 4A). The fluorescence signal in the Nimotuzumab-ICG group was detected at 2 h post-injection, peaked at 12 h post-injection, gradually decreased, and remained detected 48 h post-injection. The fluorescence signal of the Atezolizumab-Cy5.5 group was detected at 2 h post-injection and peaked at 12 h post-injection. After the simultaneous injection of Nimotuzumab-ICG and Atezolizumab-Cy5.5, both fluorescence signals were detected at 2 h and peaked at 12 h post-injection. The FMI TBR of the target group was higher than that of the corresponding block group at the corresponding time points. The difference in the TBR was more significant at 12 h than other time points. (Fig. 4B). After 48 h of in vivo observation, the tumors and major organs were dissected for ex vivo FMI. The fluorescence signal of the tumor region in the targeted group was higher than that in the blocking group. The fluorescence signals of Nimotuzumab-ICG and Atezolizumab-Cy5.5 were stronger in the liver and certain signals in the kidney, indicating that the imaging probes were mainly metabolized through the liver and some through kidney. Animal study suggested that Nimotuzumab-ICG and Atezolizumab-Cy5.5 dual-targeting imaging probes possessed good OSCC tumor-targeting capability and safety, making them suitable for further clinical application and translation.

### Identification of EGFR and PD-L1 expression on OSCC patients’ fresh biopsied samples using Nimotuzumab-ICG and Atezolizumab-Cy5.5 imaging probes

**Fig. 5:**
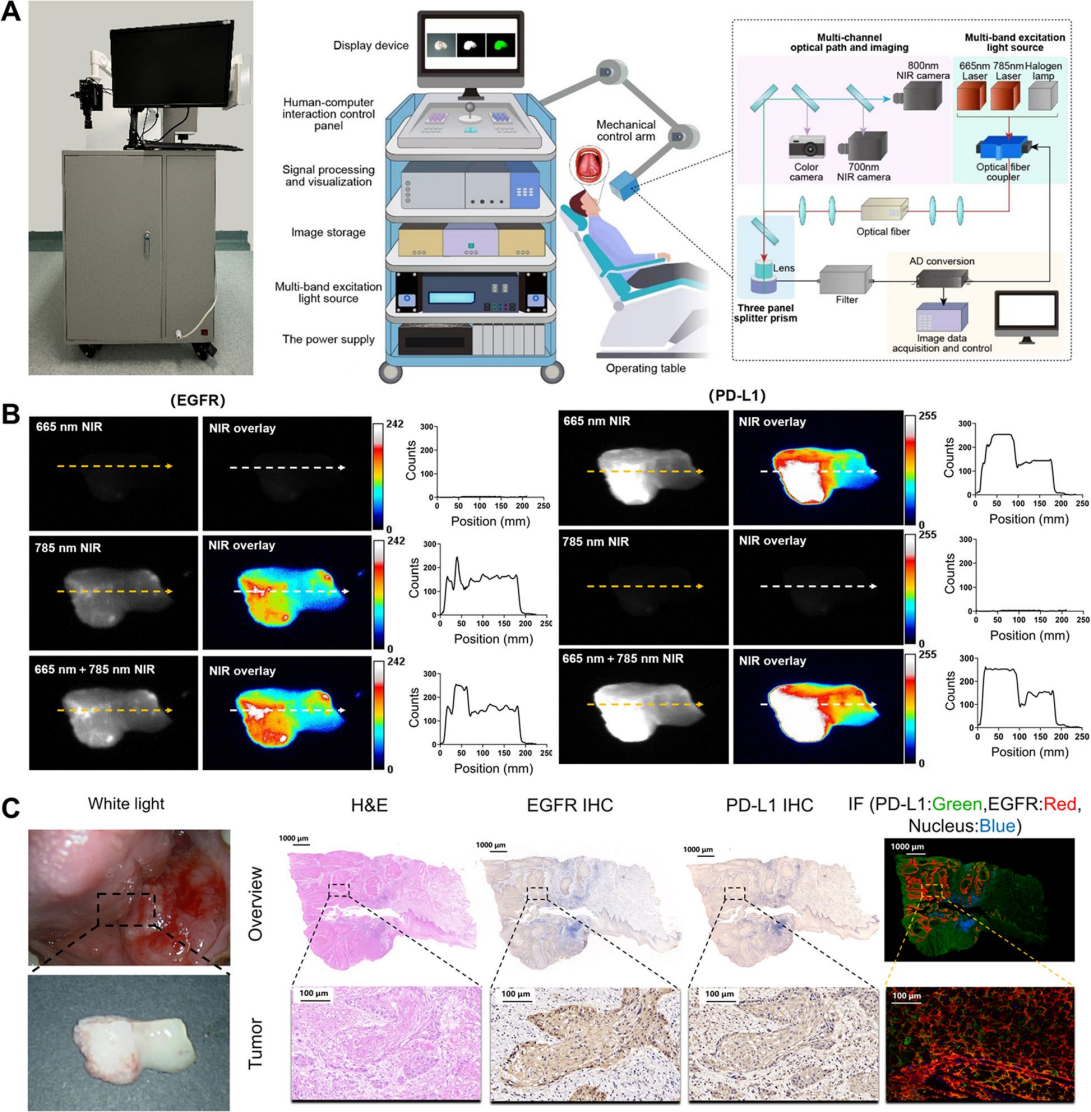
Nimotuzumab-ICG and Atezolizumab-Cy5.5 imaging probes were used to detect fresh OSCC biopsy samples. **A** The working model diagram and prototype of the home-made multispectral fluorescence imaging system. **B** Representative NIR and NIR overlay image of Nimotuzumab-ICG and Atezolizumab-Cy5.5 staining of fresh OSCC biospecimen from patients. Orange and white arrows correspond to the position and orientation of the cross-sectional fluorescence intensity profiles of the NIR and NIR overlay regions, respectively. Quantitative analysis showed that the fluorescence intensity in the tumor area and the para tumor area. NIR, Near-infrared. **C** White light, H&E, EGFR IHC, PD-L1 IHC, and IF staining of fresh biospecimen from OSCC patients. Scale bar, 1,000 μm and 100 μm

For the identification of multitarget expression, a homemade multispectral fluorescence imaging system was developed and used to conduct imaging experiments on patient specimens, and white light and multispectral fluorescence images were obtained. Figure 5A shows the working model diagram and prototype of the multispectral fluorescence imaging system. Fresh OSCC specimens from 10 patients were topically treated with Nimotuzumab-ICG and Atezolizumab-Cy5.5 with different concentrations. The optimal incubation concentration of Nimotuzumab-ICG and Atezolizumab-Cy5.5 was found to be 0.4 mg/mL (Additional file 1: Fig. S7) for 3 min (Additional file 1: Fig. S8). The obvious fluorescence signals of Nimotuzumab-ICG and Atezolizumab-Cy5.5 from the OSCC tumor region could be specifically detected, while adjacent normal areas did not show fluorescence signals (Fig. 5B). Quantitative analysis showed that Nimotuzumab-ICG imaging discriminated between tumor and para tumor. Under 785 nm NIR, the TBR of the tumor was higher than para tumor (30.05 ± 1.87 vs 20.27 ± 2.35, ** *P* < 0.01). In addition, under 665 nm + 785 nm NIR, the TBR of tumors was higher than that of para tumor (27.59 ± 1.33 vs 16.81 ± 1.39, *** *P* < 0.001). Similarly, Atezolizumab-Cy5.5 could clearly distinguish tumors from parathyroid tumors. Under 665 nm NIR and 665 nm + 785 nm NIR, the TBR of tumor and para tumor were (23.18 ± 2.52 vs 14.07 ± 1.51, ** *P* < 0.01) and (20.15 ± 1.55 vs 11.32 ± 1.02, ** *P* < 0.01), respectively. This observation was further confirmed by H&E, EGFR, and PD-L1 immunohistochemical and immunofluorescence staining of patient’s specimens. The expression levels of EGFR and PD-L1 in OSCC tumor tissues were also observed based on the fluorescence intensity produced by excitation light irradiation at 665 nm and 785 nm. The higher the fluorescence intensity, the higher were the biomarker expression levels (Fig. 5C). Therefore, we further performed multiplexed fluorescence imaging of EGFR and PD-L1 in 30 fresh OSCC patients’ specimens. FMI results, immunohistochemical staining, and immunofluorescence staining showed that the sensitivity and specificity of Nimotuzumab-ICG were 96.4% (95%*CI*: 79.8% ~ 99.8%) (27/28) and 100% (95%*CI*: 19.8% ~ 100%) (2/2), respectively. The sensitivity and specificity of Atezolizumab-Cy5.5 were 95.2% (95%*CI*: 74.1% ~ 99.7%) (20/21) and 88.9% (95%*CI*: 50.7% ~ 99.4%) (8/9), respectively. The high imaging sensitivity and specificity of the imaging probes with high safety indicate the feasibility of topical application in human patients with OSCC in situ.

### Fluorescence imaging of Nimotuzumab-ICG and Atezolizumab-ICG in OSCC patients in situ

**Fig. 6:**
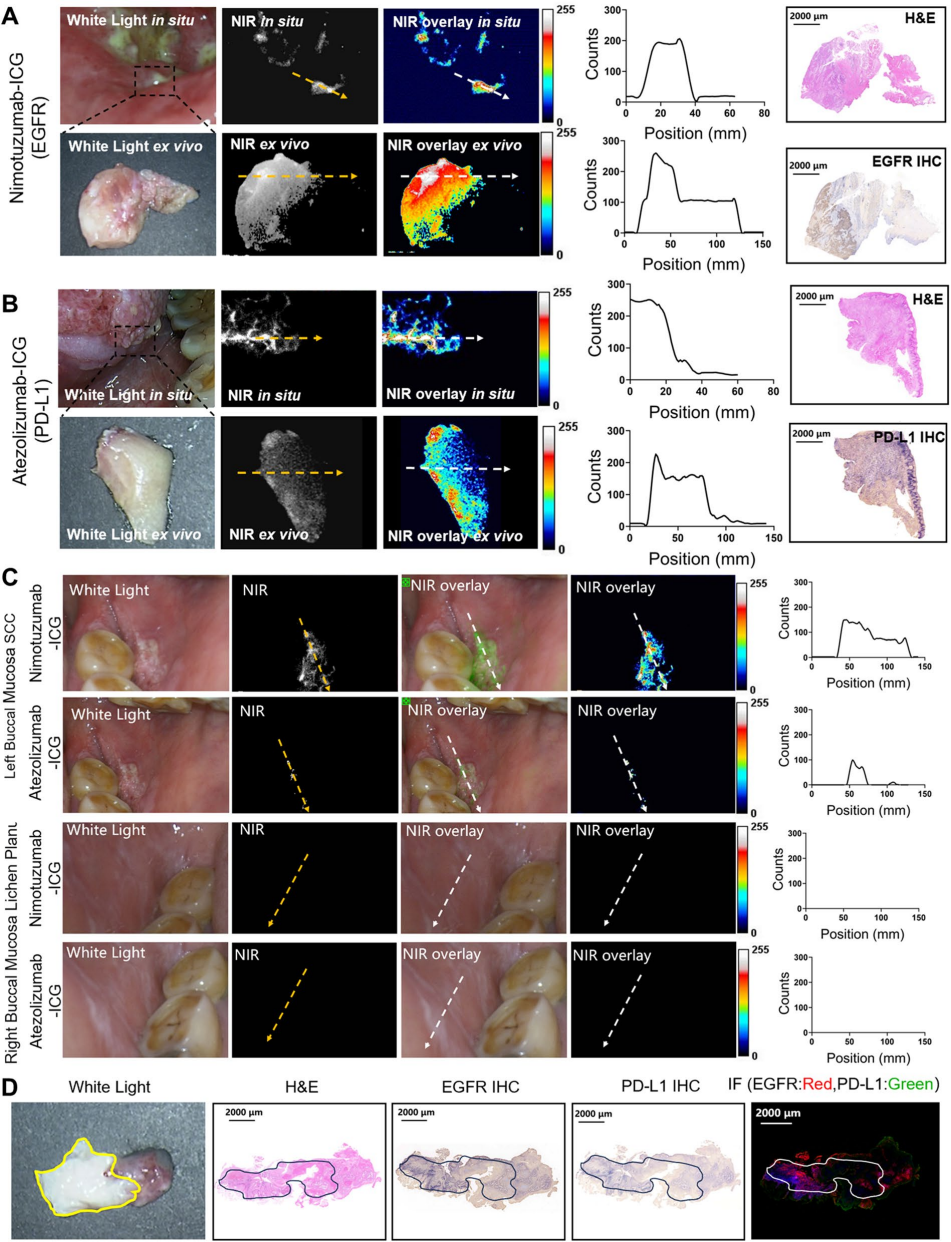
Fluorescence imaging of Nimotuzumab-ICG and Atezolizumab-ICG in OSCC human patients. **A** White light, NIR, and NIR overlay images were obtained by oral gargling experiment of Nimotuzumab-ICG on OSCC patients in clinical application. A dotted white line was drawn from tumor area to para tumor tissue to calculate the counts of fluoresce signal. The expression of EGFR was further imaged with Nimotuzumab-ICG in fresh biopsied specimens of OSCC patients, and verified using IHC and H&E staining. Scale bar, 2000 μm. **B** White light, NIR, and NIR overlay images were obtained by oral gargling experiment of Atezolizumab-ICG on OSCC patients for clinical application. The expression of PD-L1 was imaged with Atezolizumab-ICG in fresh biopsied specimens of OSCC patients, and verified using IHC. Scale bar, 2000 μm. **C** Nimotuzumab-ICG and Atezolizumab-ICG were used in the oral gargling experiment, and white light, NIR, and overlayed NIR images of both the left (with OSCC area) and right (with lichen planus) buccal mucosa were obtained. The fluorescence intensity was measured, calculated, and verified via pathology. **D** H&E, EGFR IHC, PD-L1 IHC, and IF staining were performed on fresh OSCC specimens on the patient's left buccal mucosa. The yellow line indicated the gross tumor range in fresh surgical specimens. The black line shows the tumor areas in H&E staining and IHC staining, while the white line shows the tumor areas in IF staining. Scale bar, 2,000 μm

Nimotuzumab-ICG and Atezolizumab-ICG were topically applied as mouthwashes to 8 OSCC patients with OSCC. The fluorescence imaging of EGFR and PD-L1 dual targets was mainly performed using the Digital Precision Medicine imaging device H2800 (DPM, Beijing, China), which possesses an endoscopic detector that can be applied to patients’ mouths. Quantitative analysis after gargling with Nimotuzumab-ICG (0.4 mg/ml) showed that EGFR expression was specifically highly expressed in tumor tissues but not in normal mucosa tissues (Fig. 6A, top, 23.9 ± 1.17 vs 3.01 ± 0.24, **** *P* < 0.0001). After surgical treatment, tissue specimens were subjected to ex vivo fluorescence imaging, H&E staining, and immunohistochemical staining. Quantitative analysis showed that the TBR in the tumor was higher than that in the para tumor (Fig. 6A, bottom, 48.02 ± 4.65 vs 19.27 ± 1.973, *** *P* < 0.001). Similarly, after gargling with Atezolizumab-ICG (0.4 mg/ml), quantitative analysis showed that the TBR in the tumor was higher than that in the para tumor in both in situ and ex vivo experiments. The TBR of the tumor and para tumor were 23.4 ± 2.19 vs 5.34 ± 0.61, (*** *P* < 0.001) and 27.47 ± 2.43 vs 17.06 ± 1.52 (** *P* < 0.01), respectively (Fig. 6B). The fluorescence imaging data of 8 cases of OSCC are summarized in Additional file 1: Table S3. The ex vivo FMI, HE, and IHC data were almost consistent with in vivo FMI observations, showing that specific tumor areas can be fluorescently illuminated by the targeted imaging probes.

Interestingly, a patient had buccal mucosal SCC on the left side and buccal mucosal lichen planus on the right side of the mouth. After gargling with Nimotuzumab-ICG and Atezolizumab-ICG imaging probes, fluorescence signal was observed in the left buccal mucosa, whereas no trace of fluorescence signal was observed in the right buccal mucosa (Fig. 6C). The data validated the specificity of the Nimotuzumab-ICG and Atezolizumab-ICG probes for the detection of OSCC. These in vivo observations were further verified via subsequent IHC and IF experiments (Fig. 6D).

## Discussion

Sensitive detection of tumor-specific biomarkers can facilitate the early and accurate detection of OSCC and provide guidance for precision therapy. At present, a number of studies have demonstrated the feasibility of OSCC fluorescence guided localization and guidance of surgical margin resection. Early diagnosis of OSCC was achieved through oral spray fluorescence recognition imaging [29, 30]. EGFR and PD-L1 are key targets in OSCC. Herein, we developed Nimotuzumab-ICG and Atezolizumab-Cy5.5 imaging probes combined with multispectral FMI to facilitate the noninvasive detection of EGFR and PD-L1 with high sensitivity, specificity and safety. We further translated our dual-target imaging strategy from preclinical OSCC bearing animal studies to clinical patients with OSCC, which showed great potential for the comprehensive management of OSCC for early detection and image-guided therapy, including targeted therapy, surgery, and immunotherapy.

EGFR and PD-L1 are important therapeutic targets in OSCC. EGFR, a transmembrane glycoprotein, is highly expressed in 90% of HNSCC cases and is a strong prognostic indicator of head and neck cancer [38, 39]. Maurizi et al. found that EGFR levels are associated with the risk of recurrence and death [40]. PD-L1 is also highly expressed on the surface of HNSCC [41]. The expression of PD-L1 in various solid tumors, such as esophageal, gastric, and thyroid cancers, is associated with poor prognosis [42, 43]. Bioinformatics analysis from GEPIA Datasets and immunohistochemical staining of 59 human OSCC biological specimens from the Chinese PLA General Hospital showed that EGFR and PD-L1 were highly expressed in OSCC. The dataset obtained based on GEPIA also verified that high EGFR expression in HNSCC was correlated with OS. Overexpression of PD-L1 in HNSCC is associated with DFS, not OS, which is consistent with previous findings [44]. However, whether the expression of EGFR or PD-L1 in HNSCC is correlated with OS or DFS may be subjected to differences due to the sample numbers and follow-up time. Based on a 3-year retrospective analysis of 112 patients with OSCC treated at the Chinese PLA General Hospital, a ROC curve was used to predict the clinical model. The combination analysis of EGFR and PD-L1 expression in predicting the 3-year OS of patients with OSCC can improve the prediction accuracy. Therefore, both EGFR and PD-L1 can not only be used as prognostic markers for OSCC but also as potential markers for guiding antitumor therapy.

Multiplexed FMI technology provides good opportunities for noninvasive and multitarget detection with high sensitivity and safety. A variety of malignant tumors, including OSCC, have tumor molecular heterogeneity [45], The multispectral fluorescence probes make up for the situation that the imaging of a single mode or the recognition of a single target makes the ability of tumor characterization and boundary analysis relatively challenging. And multispectral is more powerful than single spectrum in defining tumor heterogenous properties and boundary analysis [24]. And thus the detection of multitargets is most likely to improve both the sensitivity and specificity of tumor recognition and for accurate clinical diagnosis and guidance of treatment. The fluorescence-labeled anti-EGFR antibody Cetuximab-IRDye800CW has been used to locate tumors and surrounding tissues in advanced HNSCC [34]. Intravenous injection of Cetuximab-IRDye800 in patients with HNSCC achieved high sensitivity and specificity and successfully distinguished tumors from normal tissues in FMI-guided surgical studies [46, 47]. As a humanized monoclonal antibody, Nimotuzumab has a high affinity and can bind to the extracellular domain of EGFR and inhibit EGF binding [48] Nimotuzumab has been approved in several countries for the treatment of HNSCC [49] and glioma [50] based on clinical trials of various malignancies [51]. Nimotuzumab has the advantage of low toxicity compared with Cetuximab [35]. Therefore, in this study, we developed a novel EGFR targeting Nimotuzumab-ICG fluorescence imaging probe. In OSCC tumor tissues with high EGFR expression, Nimotuzumab-ICG showed a higher fluorescence imaging signal and better image contrast than images of tumors with low or no EGFR expression. Moreover, we developed an Atezolizumab-Cy5.5 fluorescence imaging probe targeting the PD-L1 molecule, which can realize noninvasive and dynamic imaging of PD-L1 expression and provide guidance for safe immunotherapy. Targeted molecular imaging of PD-L1 is an effective method for dynamic and noninvasive evaluation of PD-L1 expression; however, the main administration method is the intravenous route [37, 52–54]. In this study, we administered imaging probes through topically administration [27–32], which can achieve almost instant imaging of EGFR and PD-L1 by spraying the target area with micro-doses of fluorescent imaging probes and detecting suspected cancer lesions. Multispectral FMI imaging of Nimotuzumab-ICG and Atezolizumab-Cy5.5 is a new non-invasive, sensitive and specific way to identify tumors. Ultimately, we will be able to offer more precise and personalized treatments.

To further promote clinical translation, we applied our imaging probes as mouthwashes to 8 OSCC patients. Since the homemade multispectral fluorescence imaging system is still in the prototype stage, it cannot be used in the surgery room. Hence, we utilized the commercialized Digital Precision Medicine imaging device H2800, though this system has only one channel for detecting ICG fluorescence signal. Hence, we developed Nimotuzumab-ICG and Atezolizumab-ICG imaging probes and applied them consecutively to OSCC patients through a mouthwash. Our imaging method is quick sensitive and specific in detecting EGFR and PD-L1 expression in patients with OSCC, which was further validated by post-surgical histology and immunohistochemistry. Moreover, our imaging strategy can discriminate tumor lesions from other oral diseases, such as buccal mucosa lichen planus. For the future clinical application of homemade multispectral fluorescence imaging system can not only provide early identification and diagnosis of OSCC, but also provide guidance for clinical surgical boundary recognition and anti-tumor targeted therapy and immunotherapy. In addition, it is still necessary to further optimize the imaging algorithm and autofocus algorithm, eliminate the image distortion caused by oral haze, and continuously improve the image recognition ability.

## Conclusions

We developed a multispectral FMI imaging strategy that demonstrated the feasibility of concurrently detecting multiple tumor biomarkers and the potential for the early detection of tumors that are molecularly heterogeneous. We synthesized the multispectral fluorescence imaging probes Nimotuzumab-ICG and Atezolizumab-Cy5.5 with OSCC targeted imaging performance and good biosafety. Make up for the situation that the imaging method of a single mode or the recognition of a single target makes the ability of tumor characterization and boundary analysis relatively Challenging. Combined with the homemade multispectral fluorescence imaging system, the EGFR and PD-L1 expression can be identified and quantified from preclinical OSCC tumor-bearing mouse models to clinical OSCC patients, which facilitates early cancer detection and provide guidance for anti-tumor targeted therapy, image-guided surgery, and immunotherapy of OSCC. We demonstrated a proof-of-concept for detecting multiple targets concurrently in patients with OSCC neoplasia, which is promising for a wide range of clinical applications, including early screening and surveillance, biopsy guidance, quick staining of fresh biopsies, in vivo diagnosis, and intraoperative margin delineation.

### Supplementary Information

Additional file 1: Table S1. Demographics of OSCC patients. Table S2. Results of the Acute Oral Toxicity Test. Table S3. Clinical data of mouthwash trials for OSCC patients. Fig. S1. Synthesis of Nimotuzumab-ICG and Atezolizumab-Cy5.5. Fig. S2. Excitation and absorption spectra of Nimotuzumab-ICG and Atezolizumab-Cy5.5 under excitation light at 780 nm and 640 nm, respectively. Fig. S3. HOK, HSC3 and CAL27-fLUC cell lines were treated with cell viability at different concentrations of Nimotuzumab-ICG and Atezolizumab-Cy5.5. Fig. S4. Serum assessment of liver function markers. Fig. S5. The in vivo toxicity of Nimotuzumab-ICG and Atezolizumab-Cy5.5 were evaluated in healthy BALB/c-nu/nu male mice. H&E staining of heart, liver, kidney and spleen in different groups. Scale bar, 100 μm. Fig. S6. Bioluminescence imagingexperiments demonstrated the location of tongue tumors in a mouse model. Fig. S7. Optimal concentration analysis of Nimotuzumab-ICG and Atezolizumab-Cy5.5. Fig. S8. Analysis of optimum incubation time of Nimotuzumab-ICG and Atezolizumab-Cy5.5

Additional file 2. Original images of Western blotting

## Data Availability

The datasets used and analyzed during our study are available from the corresponding author on reasonable request.

## Abbreviations

OSCC: Oral squamous cell carcinoma
EGFR: Epidermal growth factor receptor
PD-L1: Programmed death-ligand 1
FMI: Fluorescence molecular imaging
CT: Computed Tomography
MRI: Magnetic Resonance Imaging
NIRF: Near-infrared fluorescence
ICG: Indocyanine green
HNSCC: Head and Neck squamous cell carcinoma
ROC: Receiver operator characteristic
IHC: Immunohistochemistry
H&E: Hematoxylin and eosin
ROI: Region of interest
TBR: Tumor to background ratio
