# Supplementary material for: Multispectral fluorescence imaging of EGFR and PD-L1 for precision detection of oral squamous cell carcinoma: a preclinical and clinical study
Source: BMC Med. 2024 Aug 26;22:342. doi: 10.1186/s12916-024-03559-w (PMC11346054; doi:10.1186/s12916-024-03559-w)
Supplement: Supplementary file 1 — Additional file 1: Table S1. Demographics of OSCC patients. Table S2. Results of the Acute Oral Toxicity Test. Table S3. Clinical data of mouthwash trials for OSCC patients. Fig. S1. Synthesis of Nimotuzumab-ICG and Atezolizumab-Cy5.5. Fig. S2. Excitation and absorption spectra of Nimotuzumab-ICG and Atezolizumab-Cy5.5 under excitation light at 780 nm and 640 nm, respectively. Fig. S3. HOK, HSC3 and CAL27-fLUC cell lines were treated with cell viability at different concentrations of Nimotuzumab-ICG and Atezolizumab-Cy5.5. Fig. S4. Serum assessment of liver function markers. Fig. S5. The in vivo toxicity of Nimotuzumab-ICG and Atezolizumab-Cy5.5 were evaluated in healthy BALB/c-nu/nu male mice. H&E staining of heart, liver, kidney and spleen in different groups. Scale bar, 100 μm. Fig. S6. Bioluminescence imagingexperiments demonstrated the location of tongue tumors in a mouse model. Fig. S7. Optimal concentration analysis of Nimotuzumab-ICG and Atezolizumab-Cy5.5. Fig. S8. Analysis of optimum incubation time of Nimotuzumab-ICG and Atezolizumab-Cy5.5 [file 12916_2024_3559_MOESM1_ESM.docx]

**Supplementary Data**

Multispectral fluorescence imaging of EGFR and PD-L1 for precision detection of oral squamous cell carcinoma: A preclinical and clinical study

Nenghao Jin^1,2,3,†^, Yu An^4,†^, Yu Tian^1,5,†^, Zeyu Zhang^4,†^, Kunshan He^3,7^, Chongwei Chi^3,6^, Wei Mu^4, *^, Jie Tian^3,4,*^, Yang Du^3,6,*^

1 Medical School of Chinese PLA, Beijing 100853, China

2 Department of Stomatology, The First Medical Centre, Chinese PLA General Hospital, Beijing 100853, China

3 CAS Key Laboratory of Molecular Imaging, Beijing Key Laboratory of Molecular Imaging, Institute of Automation, Chinese Academy of Sciences, Beijing, 100190, China.

4 Key Laboratory of Big Data-Based Precision Medicine (Beihang University), Ministry of Industry and Information Technology of the People’ s Republic of China, School of Engineering Medicine, Beihang University, Beijing, 100191, China.5 Beijing Hospital of Integrated Traditional Chinese and Western Medicine, Beijing 100039, China.

6 University of Chinese Academy of Sciences, Beijing 100080, China

7 State Key Laboratory of Computer Science and Beijing Key Lab of Human-Computer Interaction, Institute of Software, Chinese Academy of Sciences, Beijing 100190, China

†Nenghao Jin, Yu An, Yu Tian, Zeyu Zhang contributed equally to this work

*Correspondence should be addressed to: Yang Du: [yang.du@ia.ac.cn](mailto:yang.du@ia.ac.cn), Jie Tian: ji.tian@ia.ac.cn, Wei Mu: weimu@buaa.edu.cn

**Table S1** **Demographics of OSCC patients**

| Variable | OSCC Patients (n=112) | OSCC Patients  (n=59) |
| --- | --- | --- |
| Age (years, median) | 59.76±14.09 | 60.05±13.68 |
| Gender (n) |  |  |
| male/female | 73/39 | 39/20 |
| Location |  |  |
| Tongue | 50 | 32 |
| Buccal mucosa | 14 | 11 |
| Floor of mouth | 11 | 7 |
| Gingiva | 29 | 7 |
| Palatine | 8 | 2 |
| Tumor size stage |  |  |
| T1+T2 | 44 | 23 |
| T3+T4 | 68 | 36 |
| Nodal status stage |  |  |
| N0 | 50 | 23 |
| N1+N2 | 62 | 36 |
| Histopathological grade |  |  |
| High grade | 53 | 25 |
| Moderate grade | 39 | 22 |
| Low grade | 20 | 12 |

**Table S2 Results of the Acute Oral Toxicity Test**

| Sample | Gender | Grouping of Doses  (mg/kg) | Size of Animal | Manifestation of Toxicity | Number of Animals Killed | Death Rate  (%) |
| --- | --- | --- | --- | --- | --- | --- |
| Nimotuzumab-ICG | ♀ | 5000 | 10 | None | 0 | 0 |
|  | ♂ | 5000 | 10 | None | 0 | 0 |
| Atezolizumab-ICG | ♀ | 5000 | 10 | None | 0 | 0 |
|  | ♂ | 5000 | 10 | None | 0 | 0 |

**Table** **S3 Clinical data of mouthwash trials for OSCC patients**

| ID | Gender | Age | Disease Locations | Histopathological Grade | EGFR | | PD-L1 | |
| --- | --- | --- | --- | --- | --- | --- | --- | --- |
|  |  |  |  |  | Spray Nimotuzumab-ICG Imaging | IHC Staining | Spray Atezolizumab-ICG Imaging | IHC Staining |
| 1 | Female | 54 | Left Buccal | High Grade | ＋ | ＋ | － | － |
| 2 | Female | 57 | Left Tongue | High Grade | ＋ | ＋＋ | ＋ | － |
| 3 | Male | 50 | Right Floor of Mouth | High Grade | ＋ | ＋ | ＋＋ | ＋＋ |
| 4 | Female | 76 | Left Buccal | High Grade | ＋ | ＋ | － | － |
| 5 | Female | 76 | Left Tongue | Moderate Grade | ＋ | － | ＋ | ＋ |
| 6 | Male | 53 | Right Tongue | Moderate Grade | ＋ | ＋ | ＋ | ＋＋ |
| 7 | Male | 82 | Left Tongue | Low Grade | ＋＋ | ＋ | ＋ | ＋ |
| 8 | Female | 66 | Left Floor of Mouth | Low Grade | ＋＋ | ＋＋＋ | ＋ | ＋ |


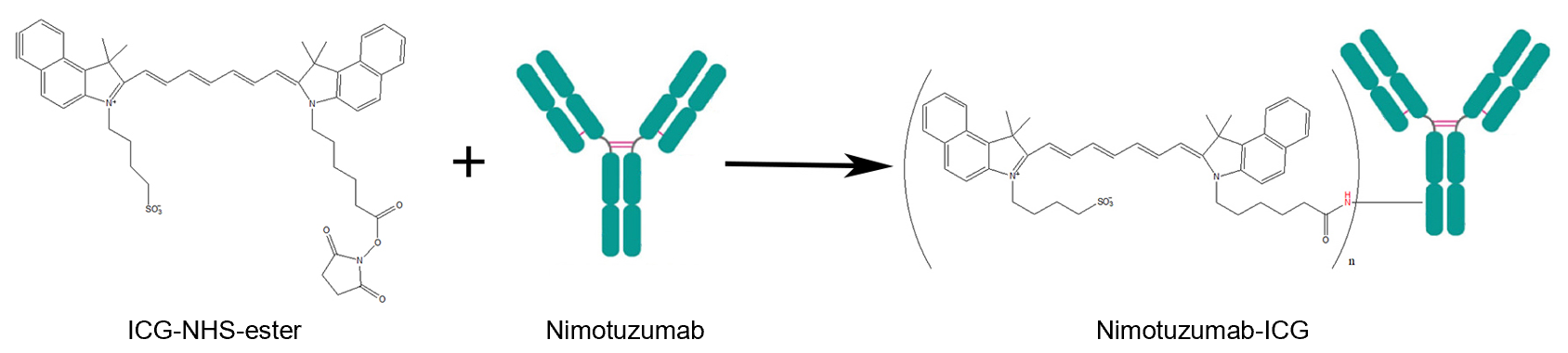


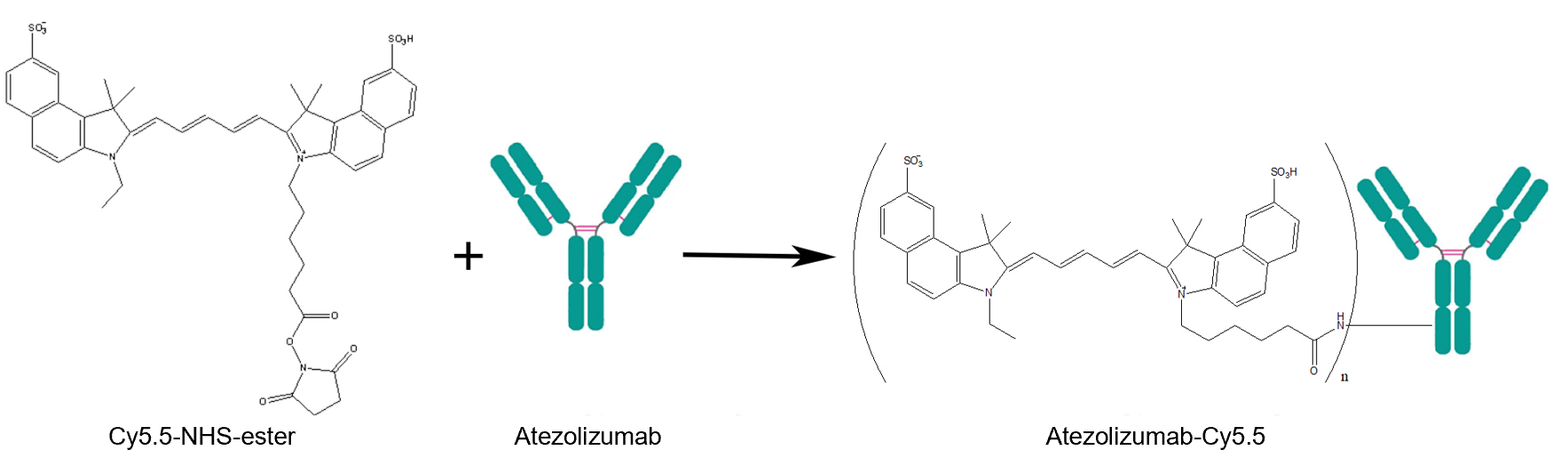


**Fig. S1 Synthesis of Nimotuzumab-ICG and Atezolizumab-Cy5.5.**


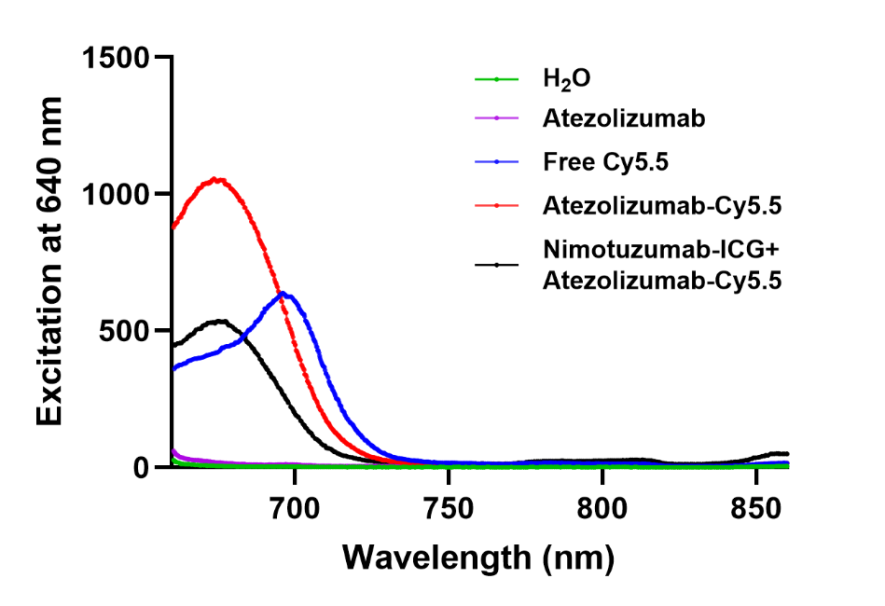

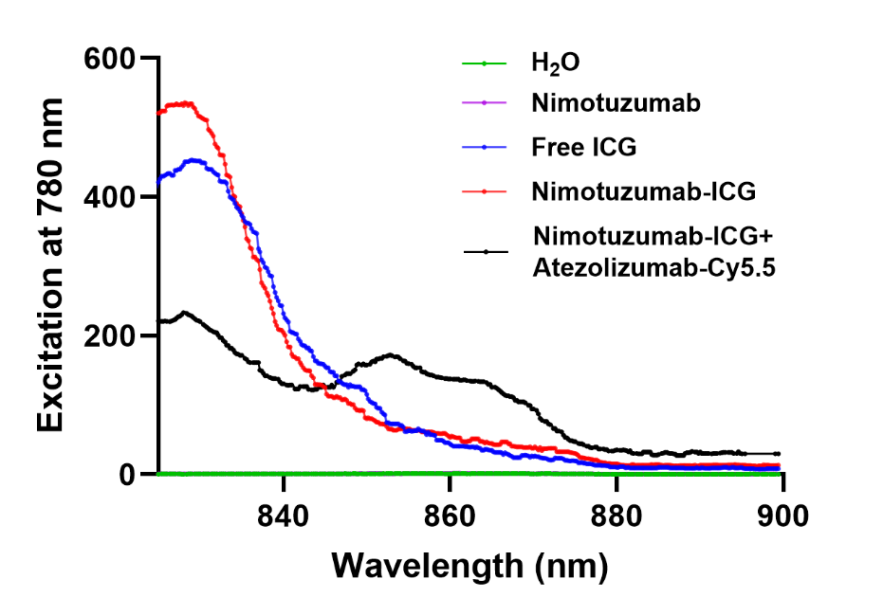

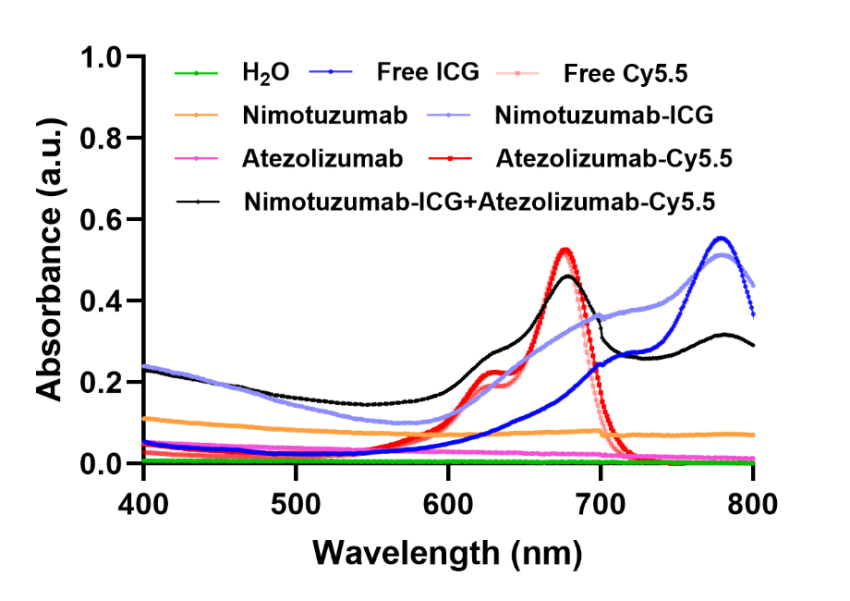


**Fig. S2 Excitation and absorption spectra of Nimotuzumab-ICG and Atezolizumab-Cy5.5 under excitation light at 780 nm and 640 nm, respectively.**


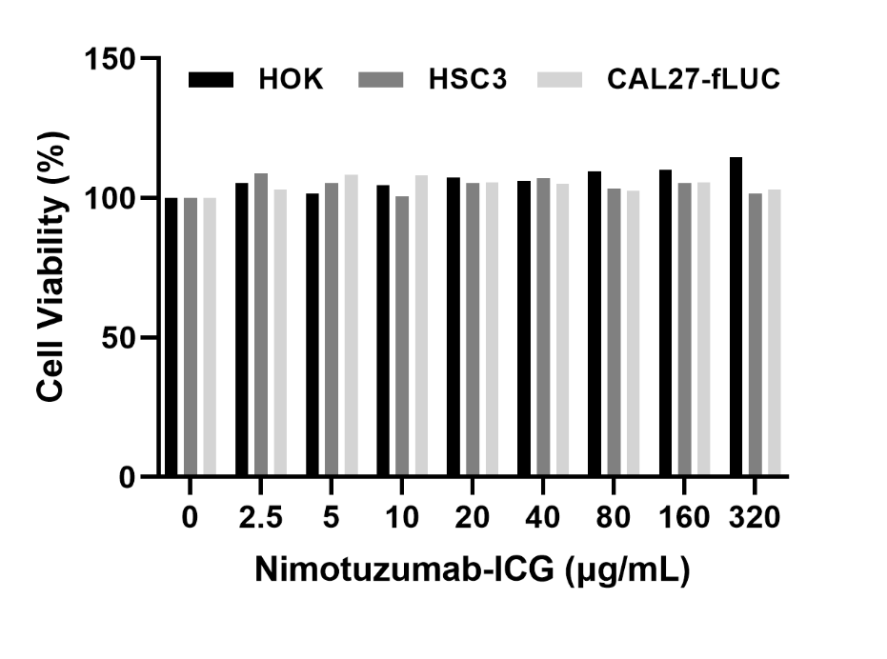

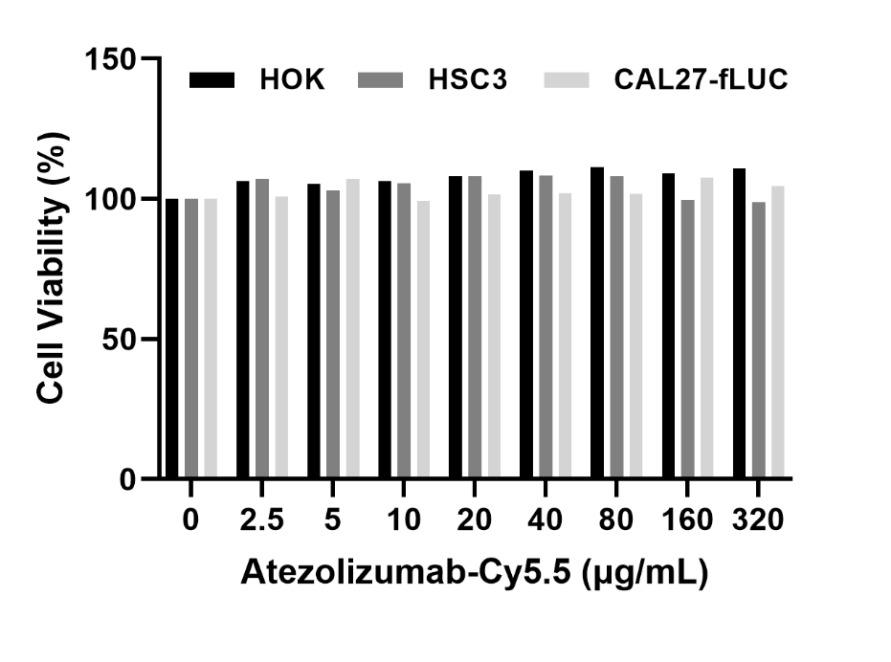

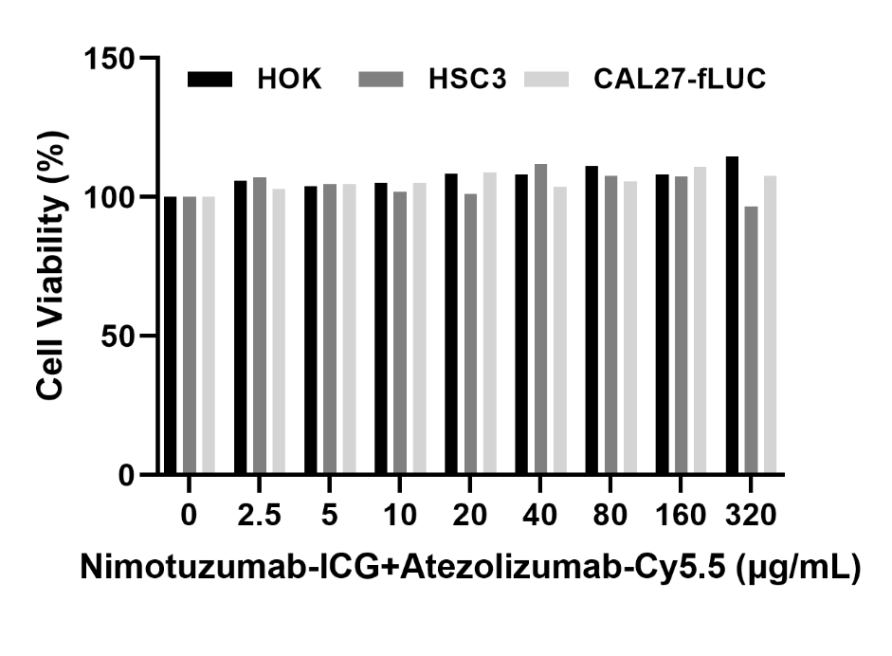


**Fig. S3** **HOK, HSC3 and CAL27-fLUC cell lines were treated with cell viability at different concentrations of Nimotuzumab-ICG and Atezolizumab-Cy5.5.**


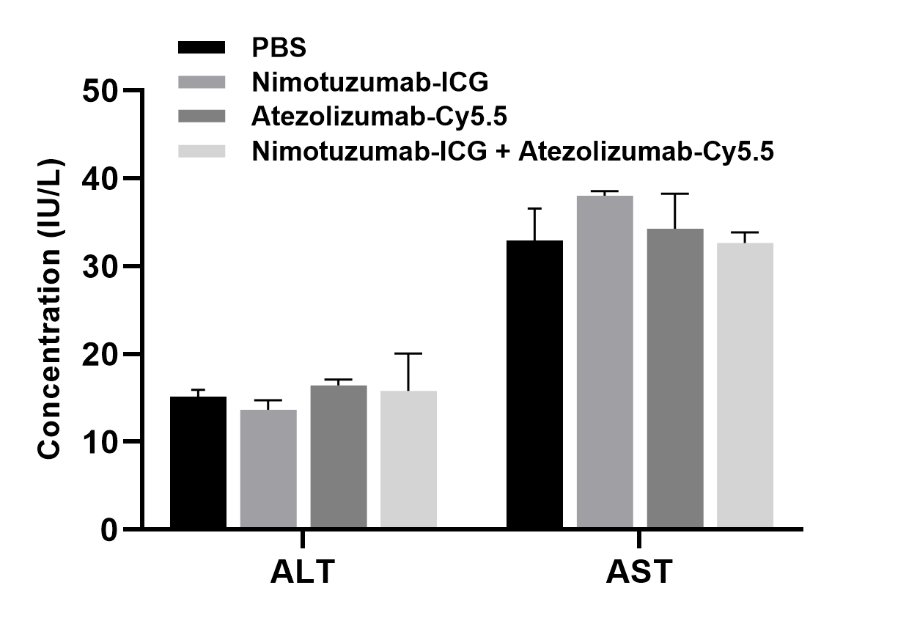


**Fig. S4 Serum assessment of liver function markers.**

**
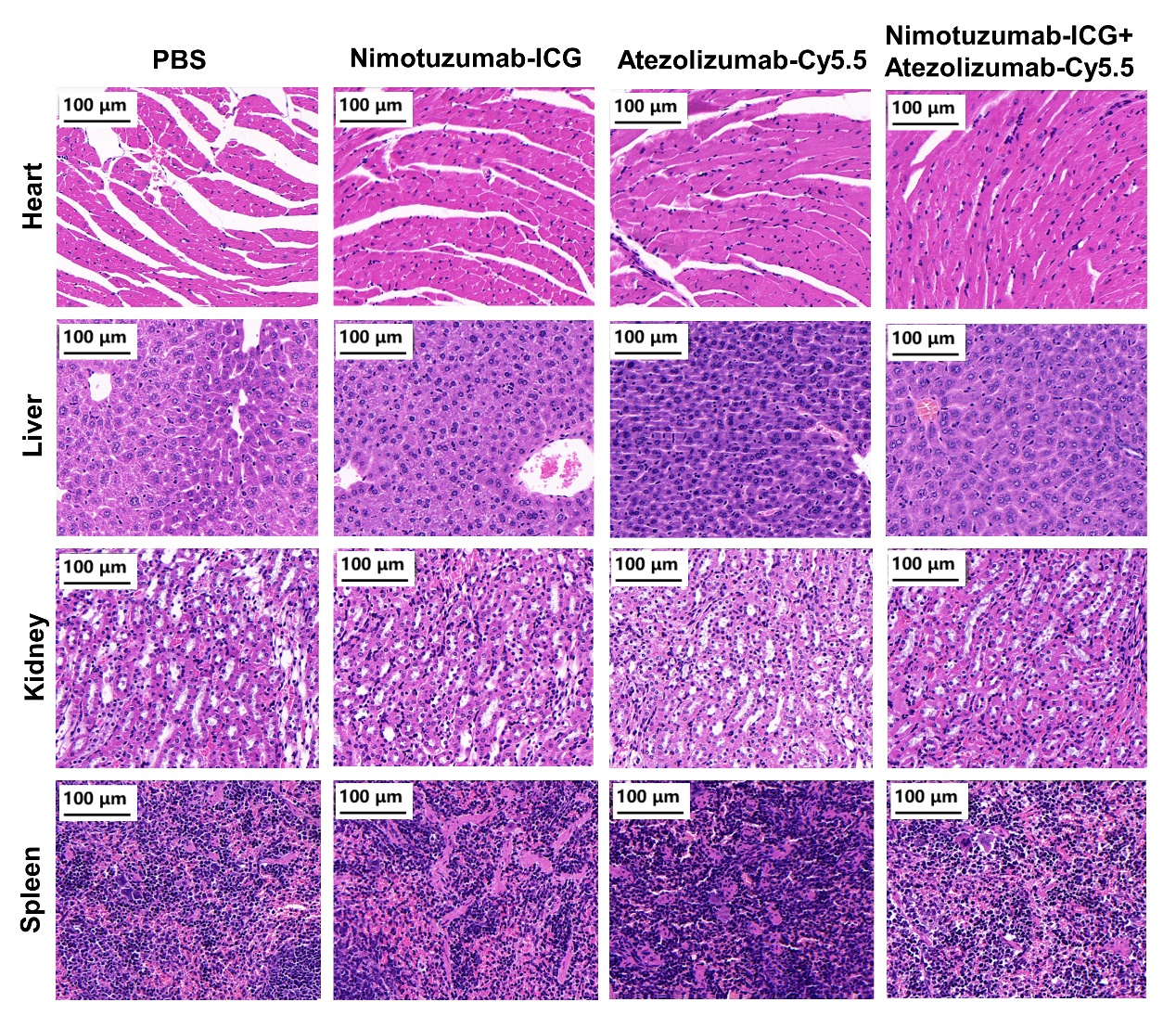
**

**Fig. S5** **The *in vivo* toxicity of Nimotuzumab-ICG and Atezolizumab-Cy5.5 were evaluated in healthy BALB/c-nu/nu male mice**. H&E staining of heart, liver, kidney and spleen in different groups. Scale bar, 100 μm.


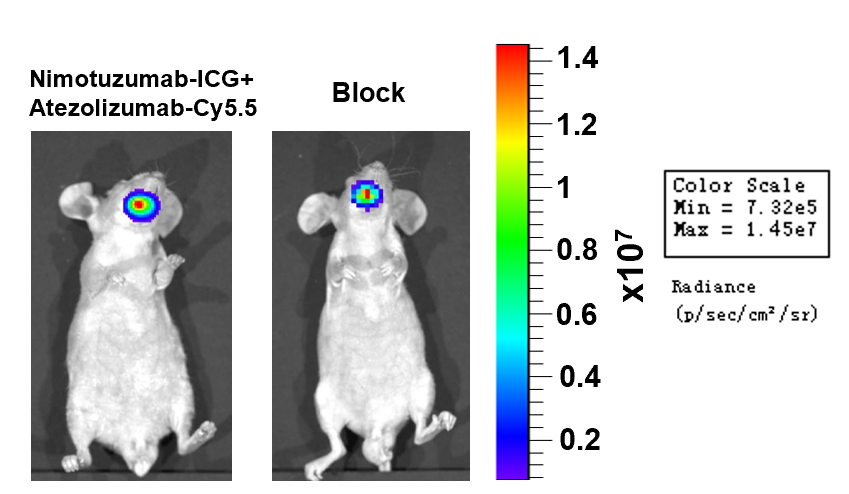


**Fig. S6** **Bioluminescence imaging (BLI) experiments demonstrated the location of tongue tumors in a mouse model.**


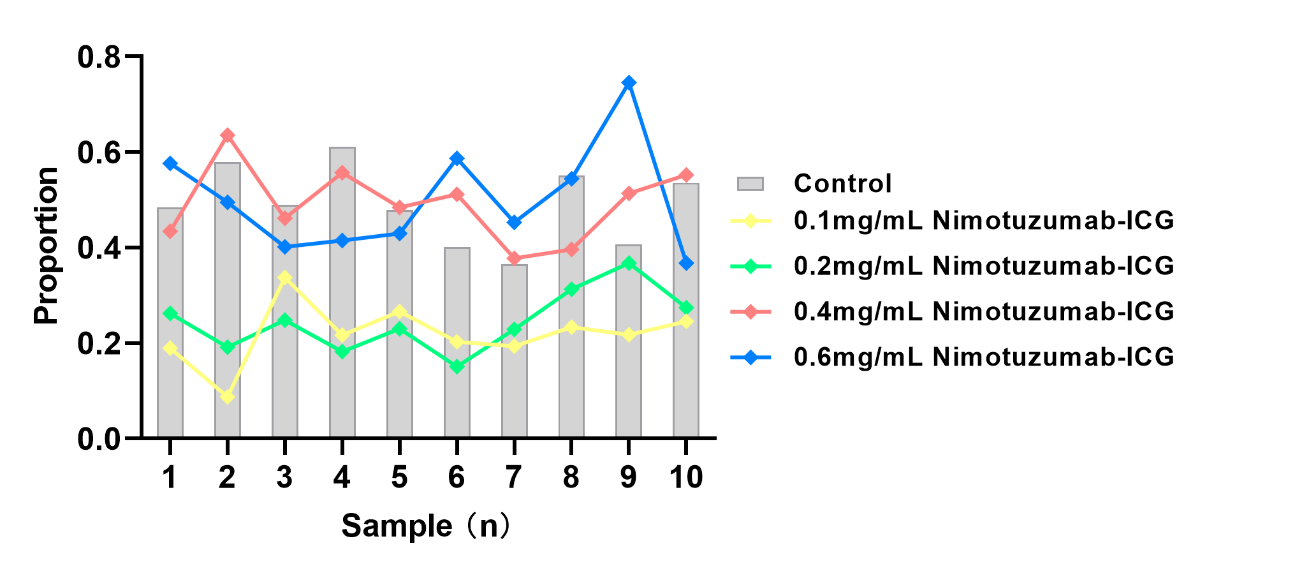

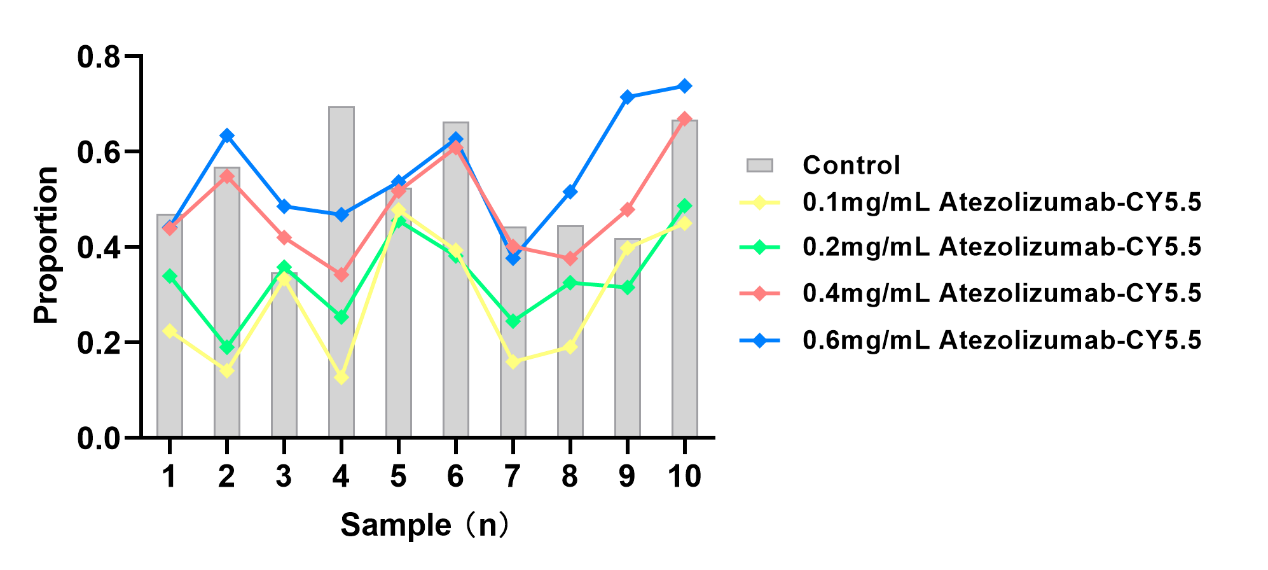


**Fig. S7** **Optimal concentration analysis of Nimotuzumab-ICG and Atezolizumab-Cy5.5.**

**
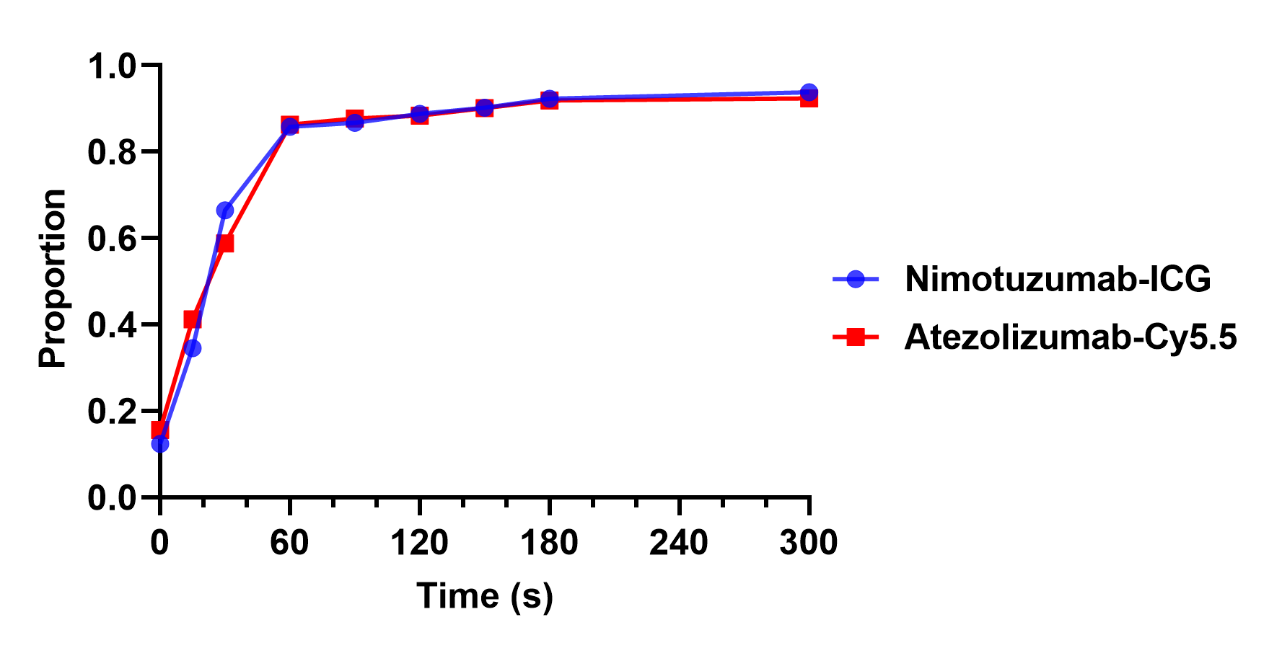
**

**Fig. S8** **Analysis of optimum incubation time of Nimotuzumab-ICG and Atezolizumab-Cy5.5**
