# Supplementary material for: Multispectral fluorescence imaging of EGFR and PD-L1 for precision detection of oral squamous cell carcinoma: a preclinical and clinical study
Source: BMC Med. 2024 Aug 26;22:342. doi: 10.1186/s12916-024-03559-w (PMC11346054; doi:10.1186/s12916-024-03559-w)

Western blotting 1

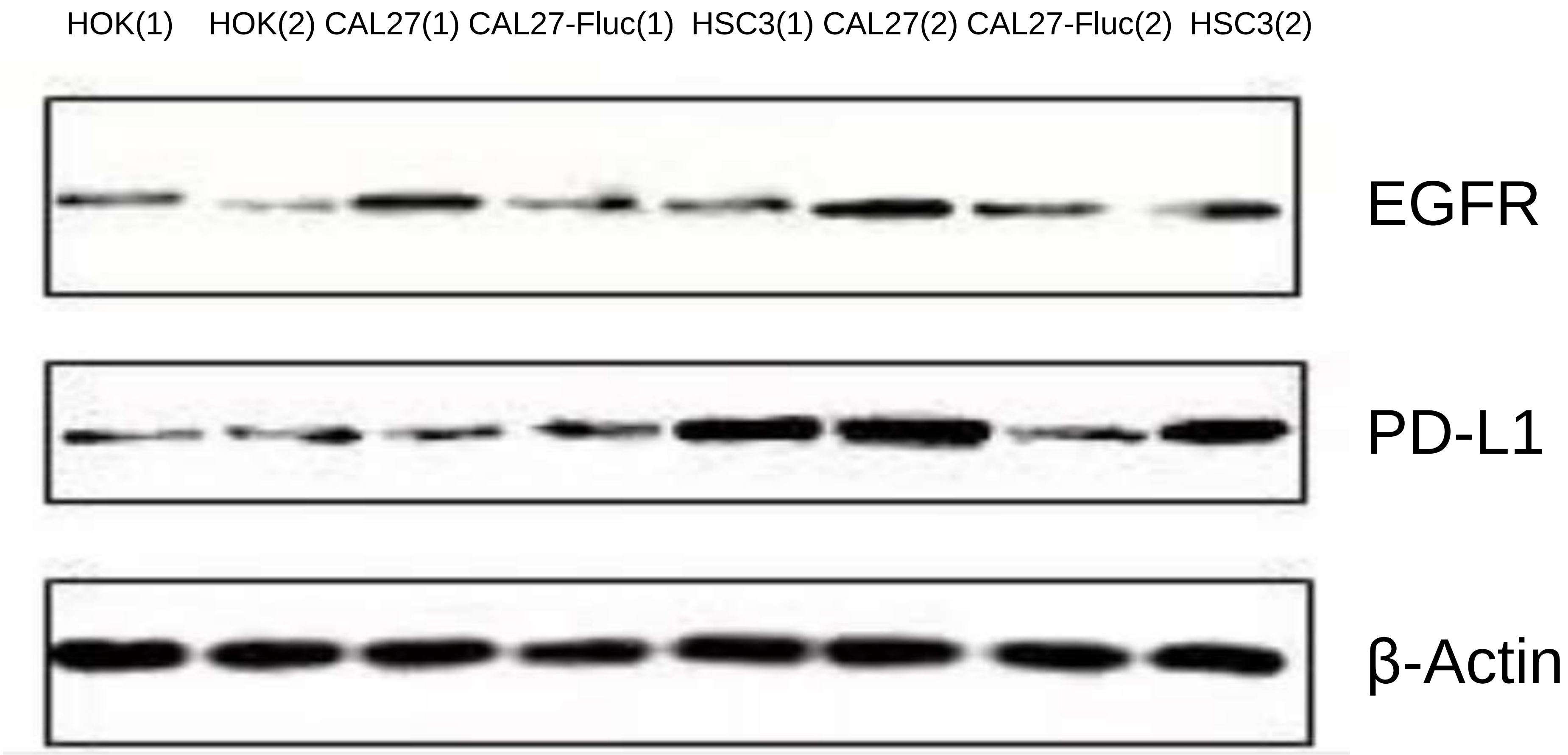

Western blotting 2

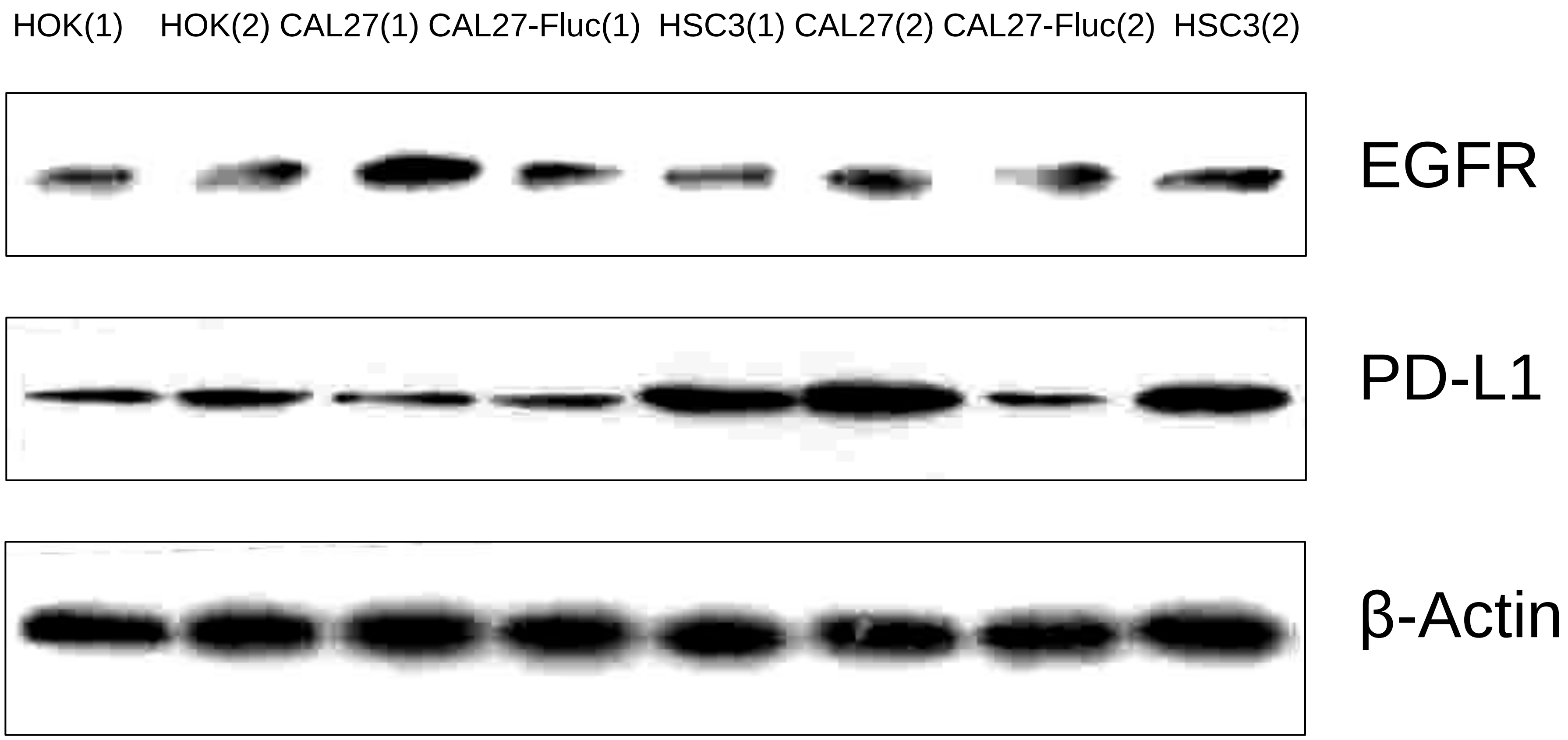

Western blotting 2

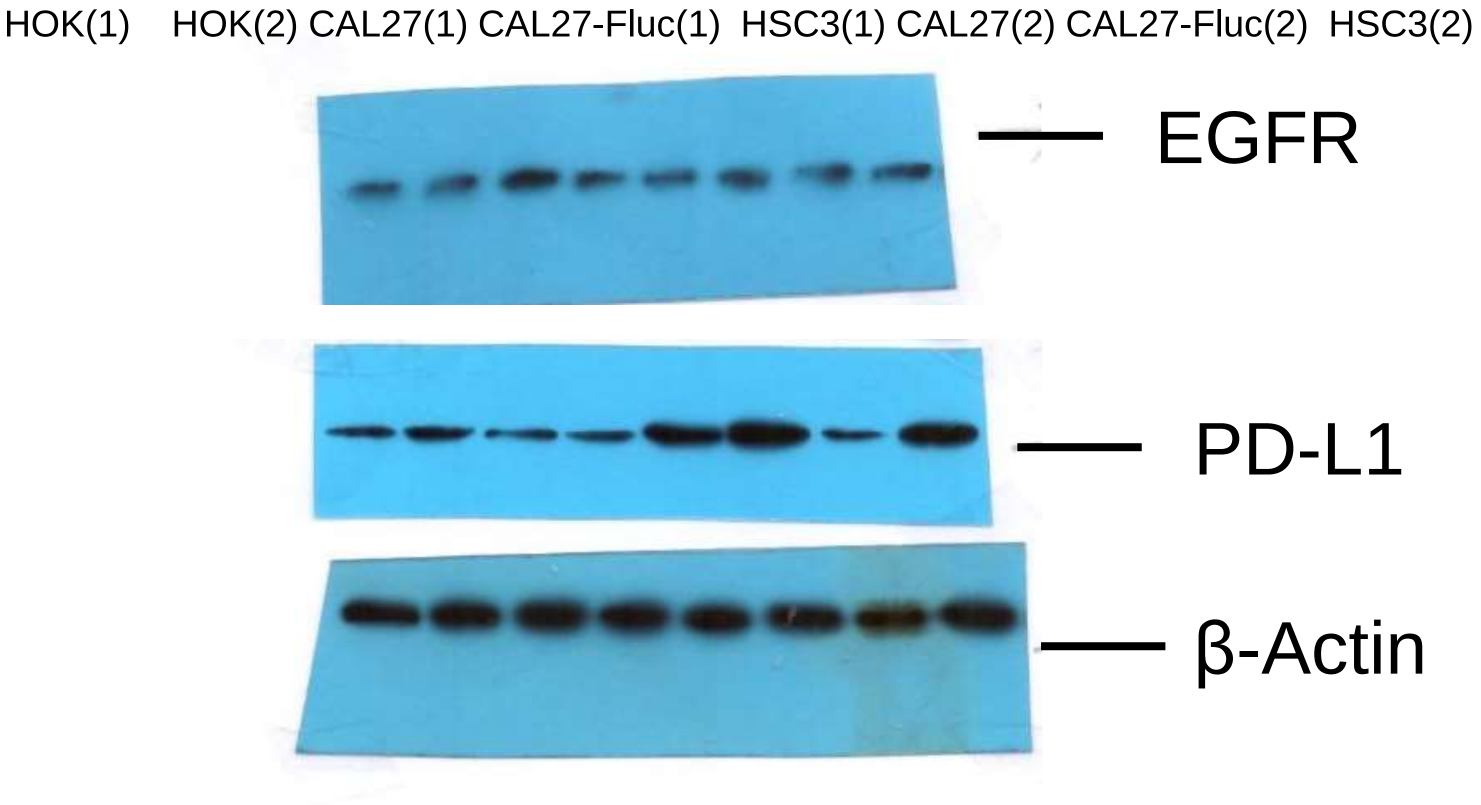

Supplement: Supplementary file 2 — Additional file 2. Original images of Western blotting [file 12916_2024_3559_MOESM2_ESM.pdf]
